# Infection with genotoxin‐producing *Salmonella enterica* synergises with loss of the tumour suppressor *APC* in promoting genomic instability via the PI3K pathway in colonic epithelial cells

**DOI:** 10.1111/cmi.13099

**Published:** 2019-08-26

**Authors:** Océane C.B. Martin, Anna Bergonzini, Federica D'Amico, Puran Chen, Jerry W. Shay, Jacques Dupuy, Mattias Svensson, Maria G. Masucci, Teresa Frisan

**Affiliations:** ^1^ Department of Cell and Molecular Biology Karolinska Institutet Stockholm Sweden; ^2^ Department of Molecular Biology Umeå University Umeå Sweden; ^3^ Department of Medicine Karolinska Institutet Stockholm Sweden; ^4^ Department of Cell Biology The University of Texas Southwestern Medical Center Dallas Texas USA; ^5^ INRA, ToxAlim (Research Centre in Food Toxicology), INRA, ENVT, INP‐Purpan, UPS Université de Toulouse Toulouse France

**Keywords:** APC, bacteria and cancer, bacterial genotoxin, DNA damage response, DNA repair, organotypic model, tumour‐suppressor gene

## Abstract

Several commensal and pathogenic Gram‐negative bacteria produce DNA‐damaging toxins that are considered bona fide carcinogenic agents. The microbiota of colorectal cancer (CRC) patients is enriched in genotoxin‐producing bacteria, but their role in the pathogenesis of CRC is poorly understood. The *adenomatous polyposis coli* (*APC*) gene is mutated in familial adenomatous polyposis and in the majority of sporadic CRCs. We investigated whether the loss of *APC* alters the response of colonic epithelial cells to infection by *Salmonella enterica*, the only genotoxin‐producing bacterium associated with cancer in humans. Using 2D and organotypic 3D cultures, we found that APC deficiency was associated with sustained activation of the DNA damage response, reduced capacity to repair different types of damage, including DNA breaks and oxidative damage, and failure to induce cell cycle arrest. The reduced DNA repair capacity and inability to activate adequate checkpoint responses was associated with increased genomic instability in APC‐deficient cells exposed to the genotoxic bacterium. Inhibition of the checkpoint response was dependent on activation of the phosphatidylinositol 3‐kinase pathway. These findings highlight the synergistic effect of the loss of APC and infection with genotoxin‐producing bacteria in promoting a microenvironment conducive to malignant transformation.

## 1 INTRODUCTION

Colorectal cancer (CRC) is the third most common cancer (IARC, n.d.) and a leading cause of cancer death (reviewed in Siegel, Desantis, & Jemal, 2014). Approximately 15% to 30% of CRCs occur within families, and the occurrence of germline mutations in the hereditary nonpolyposis colorectal cancer syndromes and familial adenomatous polyposis (FAP) has been extensively studied. All FAP patients carry inactivating mutations of the *adenomatous polyposis coli* (*APC*) tumour suppressor gene, and the gene is also mutated in 70–80% of sporadic colorectal adenomas and carcinomas (reviewed in Fearon, 2011). In addition to germline genetic alterations, different diets and lifestyles have a significant impact on colorectal carcinogenesis (reviewed in Huxley et al., 2009). The introduction of next generation sequencing has highlighted significant alterations in the intestinal microbiota of CRC patients compared with healthy subjects (Villeger et al., 2018). However, it is still unknown whether the dysbiosis precedes or is a consequence of the tumour microenvironment and whether microbial products contribute to the carcinogenic process. An emerging property that may link the microbiota with the acquisition of carcinogenic traits is the enrichment in the CRC microbiome of bacterial families that produce genotoxins with the capacity to induce damage in the host cell DNA (reviewed in Villeger et al., 2018).

Three bacterial genotoxins, the cytolethal distending toxin (CDT), the typhoid toxin (TT), and colibactin (Guerra, Cortes‐Bratti, Guidi, & Frisan, 2011; Haghjoo & Galan, 2004) have been functionally characterised. CDT and TT are protein toxins that share the same CdtB active subunit, which is structurally and functionally homologue to the mammalian DNase I (Song, Gao, & Galan, 2013). The delivery of CdtB to the nucleus of intoxicated cells is accompanied by the induction of DNA single‐ and double‐strand breaks and triggering of an ATM‐dependent DNA damage response (DDR) characterised by the formation of nuclear foci of phosphorylated histone 2AX (γH2AX), relocalisation of the Mre11/Nbs1/Rad50 complex, and activation of CHK2 and p53 (Guerra, Guidi, et al., 2011; Spano, Ugalde, & Galan, 2008). As a consequence of DDR activation, the intoxicated cells are arrested in the G1 and/or G2 phases of the cell cycle, and upon failure to repair, the damage undergoes senescence or apoptosis in a cell type‐dependent manner (Blazkova et al., 2010; Guerra, Cortes‐Bratti, et al., 2011). However, intoxicated cells may occasionally survive and overcome the DDR‐induced tumorigenic barrier, leading to genomic instability and the acquisition of carcinogenic traits (Guidi, Levi, et al., 2013; Hanahan & Weinberg, 2011). Thus, the genotoxic activity of these bacterial effectors may contribute to CRC development.

To address this issue, we have assessed the consequences of infection with *Salmonella enterica*, the only genotoxin‐producing bacterium associated with an increased risk of developing hepatobiliary and colon carcinoma in humans (Dutta, Garg, Kumar, & Tandon, 2000; Mughini‐Gras et al., 2018) on alteration of the DDR and acquisition of genomic instability in normal and APC‐deficient cells, using classical two‐dimensional (2D) and organotypic three‐dimensional (3D) tissue models. We found that infection with genotoxin‐producing *Salmonella* synergises with the loss of APC to enhance genomic instability both in 2D and 3D cultures via activation of the phosphoinositide 3‐kinase (PI3K) pathway. This effect was associated with impairment of DNA repair and failure to achieve efficient cell cycle arrest in cells exposed to the DNA damage‐inducing bacterium. The latter feature was enhanced in cells grown in 3D culture, suggesting that this more complex culture setting may reveal new features of the cellular responses to DNA damage.

## 2 RESULTS

### 2.1 APC deficiency alters the DDR of cells infected with genotoxic *Salmonella*

**Figure 1:**
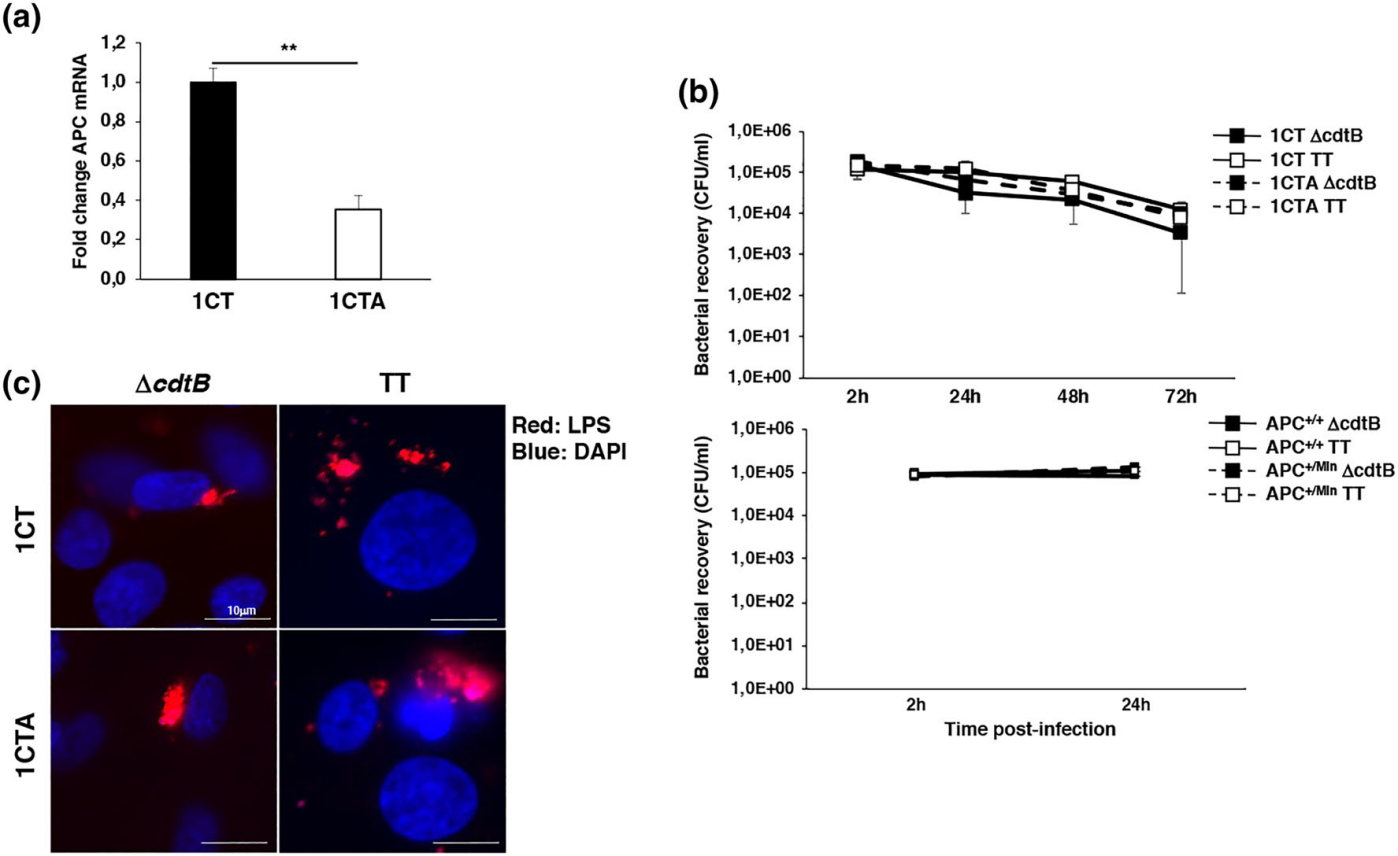
Infection with the *Salmonella* MC1 TT and MC1Δ*cdtB* strains. (a) Levels of APC mRNA assessed by qPCR in the 1CT and 1CTA cell lines. Mean ± SEM of three independent experiments. (b) 1CT and 1CTA cells (upper panel) and *Apc* ^+/+^ and *Apc* ^+/Min^ cells (lower panel) grown in 2D culture were infected with the MC1 ∆*cdtB* (∆*cdtB*) or MC1 TT (TT) strains at MOI 100:1. The data are presented as number of CFU/ml of viable bacteria recovered at the indicated time points. Mean ± SEM of four to eight independent experiments. (c) Cells grown in 2D culture were infected with the MC1 ∆*cdtB* or MC1 TT strains at MOI 100:1. Bacteria were visualised using a rabbit serum anti‐LPS followed by a goat anti‐rabbit secondary antibody conjugated to Alexa‐568 (red). Nuclei were counterstained with DAPI (blue). Representative scanning confocal micrographs at magnification 63×

Two cell models were used to investigate whether APC deficiency alters the outcome of infection with genotoxin‐producing bacteria in eukaryotic cells: (a) the immortalized non transformed human colonic epithelial cell line 1CT that carries a functional *APC* gene (Roig et al., 2010) and the isogenic 1CTA cell line where a three‐fold downregulation of the APC mRNA was obtained by stable transfection of specific shRNA (Graillot et al., 2016; Figure 1a); and (b) the SV40 large T antigen immortalized murine colonic epithelial cells that are either APC wild type (*Apc* ^*+/+*^) or APC haploinsufficient due to an allelic mutation leading to expression of a truncated inactive product (*Apc* ^*+/Min*^; Forest, Clement, Pierre, Meflah, & Menanteau, 2003). In both cell types APC deficiency was associated with disruption of actin stress fibres (Figure S1A) and increased nuclear size (Figure S1B).

Cells grown in standard 2D cultures were infected with *S*. *typhimurium* strain MC1 expressing a functional typhoid toxin (MC1 TT) or an isogenic strain that lacks the genotoxic activity due to deletion of the gene encoding for the active subunit (MC1 Δ*cdtB*) at a multiplicity of infection (MOI) of 100:1. Downregulation of the APC mRNA or APC haploinsufficiency did not influence *Salmonella* infection as assessed by the similar recovery of viable bacteria after infection (Figure 1b), and the presence of *Salmonella*‐containing vacuoles within the infected cells, detected by LPS staining, 24‐hr postinfection (Figure 1c). As expected, only infection with the MC1 TT strain induced DNA damage as assessed by a significant increase in the percentage of cells expressing early (KAP1 phosphorylation) and late (CHK2 and p53 phosphorylation) markers of DDR activation (Figures S2), therefore, the data relative to infection with the control MC1 Δ*cdtB* strain will not be included in the subsequent figures.

**Figure 2:**
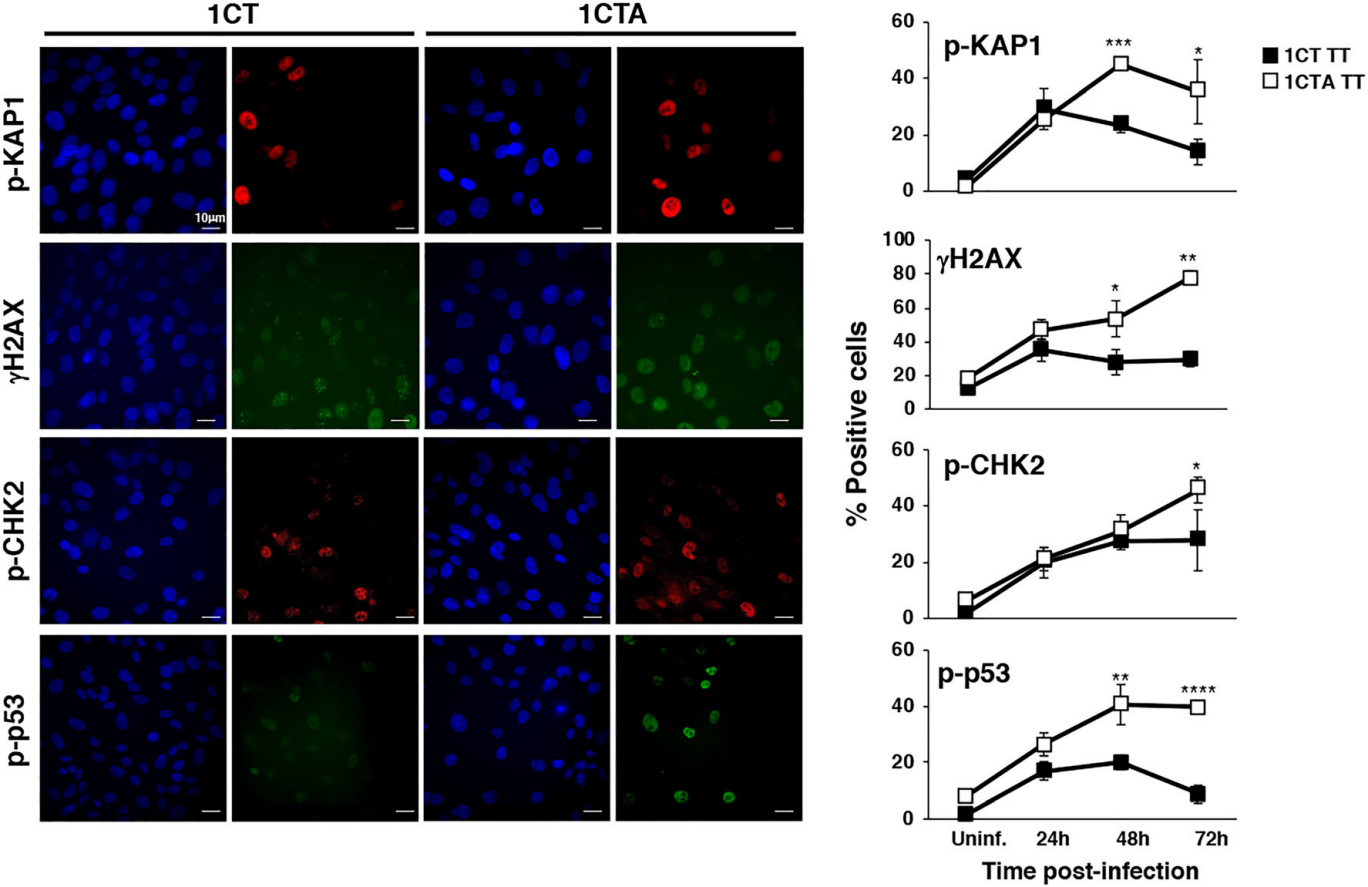
APC‐deficient cells show a sustained activation of the DNA damage response. 1CT and 1CTA cells, grown in 2D culture, were left untreated (Uninf.) or infected with the MC1 TT strain (TT) at MOI 100:1 for the indicated period of time. Activation of the DNA damage response was assessed by immunofluorescence analysis, using antibodies specific for phosphorylated KAP1 (p‐KAP1), phosphorylated H2AX (γH2AX), phosphorylated CHK2 (p‐CHK2), Ser15 phosphorylated p53 (p‐p53). Left panel: representative fluorescence micrographs; right panel: quantification of the positive cells. The percentage of positive cells in uninfected cells was similar during all the time kinetics experiment; thus, we have presented the values relative to the 24‐hr time point. Mean ± SEM of three independent experiments. One hundred cells were evaluated for each experiment for each cell line. Statistical analysis comparing 1CT and 1CTA for each time point was performed using the Student *t* test, *p < .05; **p < .01; ***p < .001; ****p < .0001

We next assessed whether APC deficiency was associated with altered activation of the DDR upon infection with the genotoxic *Salmonella*. To this end, 1CT and 1CTA cells were infected with the MC1 TT strain, and the kinetics of expression of DNA damage sensor proteins (phospho‐KAP1 and γH2AX) and transducer effectors (phospho‐CHK2 and phospho‐p53 on Ser 15) were monitored over a period of 72 hr. Infection with the genotoxic *Salmonella* caused an increase of p‐KAP1, γH2AX, p‐CHK2, and p‐p53 Ser15 positive cells in both APC‐proficient and APC‐deficient cells within the first 24 hr after infection (Figure 2). However, whereas the levels of these proteins decreased or remained constant over time in the 1CT cells, the percentage of cells positive increased over time in the 1CTA cell line for most of the markers (Figure 2). The different kinetics profile of the DDR response was not dependent on a reduced proliferation rate of the 1CTA cells compared with the APC proficient line, as shown by the growth curve presented in Figure S3A.

Similar results were observed when comparing the kinetics of the DDR activation in the *Apc* ^*+/+*^ and *Apc* ^*+/Min*^ cells (Figure S4A).

Collectively, these data indicate that APC deficiency is associated with an altered response to the *Salmonella*‐induced DNA damage, and the sustained DDR suggests a reduced capacity of the 1CTA cells to repair the damaged DNA.

### 2.2 APC deficiency is associated with reduced DNA damage repair capacity

**Figure 3:**
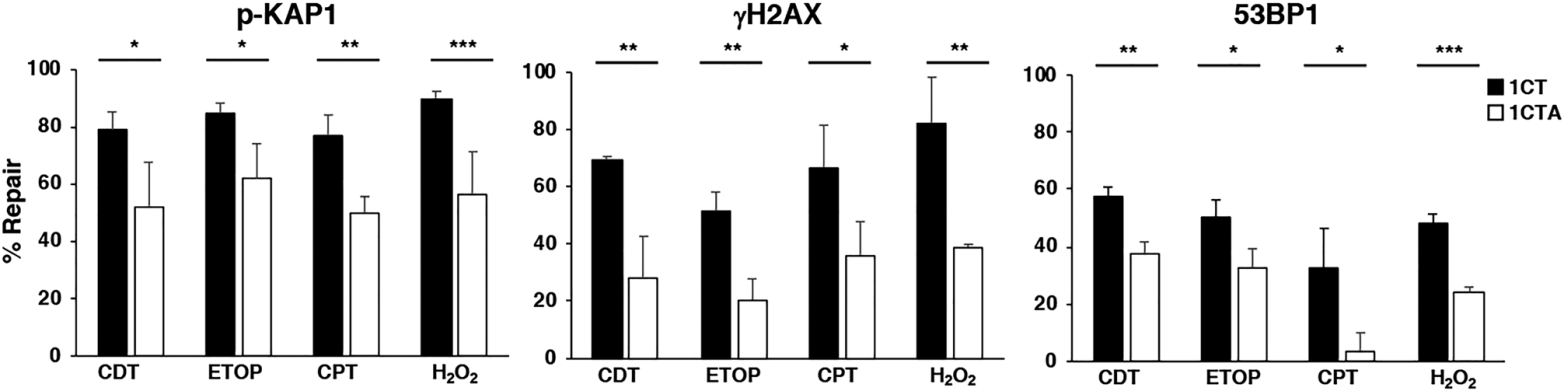
APC deficiency reduces the repair of DNA damage induced by a broad spectrum of genotoxic stresses. 1CT and 1CTA cells, grown in 2D culture, were left untreated (see Figure S5) or treated for 6 hr with CDT (1 μg/ml), etoposide (ETOP, 15 μM), camptothecin (CPT, 5 μM), or H_2_O_2_ (50 μM), defined as 6 hr. After 6‐hr incubation, cells were washed and maintained in medium without the genotoxic agents for additional 18 hr, defined as 24 hr. The percentage of repair was calculated as follows: [1 − (% positive cells at 24 hr/% positive cells at 6 hr)] × 100. One hundred cells were evaluated for each experiment for each cell line. Mean ± SEM of three independent experiments. Statistical analysis was performed using the Student *t* test, *p < .05; **p < .01; ***p < .001

To address the effect of APC deficiency on the DNA repair capacity, 1CT and 1CTA cells were exposed to a panel of DNA‐damaging agents known to cause DNA strand breaks (CDT, etoposide, and camptothecin) and oxidative damage (H_2_O_2_). The treated cells were either fixed after 6 hr to assess the extent of DNA damage or further incubated for 18 hr in fresh medium without the compounds to evaluate the residual damage by monitoring KAP1 and H2AX phosphorylation and the formation of 53BP1 foci. As shown in Figure S5, a similar response to the different DNA‐damaging agents was observed for all the three markers analysed 6‐hr posttreatment independently of the levels of APC expression. However, for all the genotoxic agents, the extent of repair was significantly lower in the 1CTA cells (Figure 3), indicating that APC deficiency reduces the capacity to repair the DNA damage induced by a broad spectrum of damaging agents. A comparable reduced capacity to repair DNA damage was obtained upon exposure of the APC haploinsufficient murine cells to etoposide, camptothecin, and H_2_O_2_ (Figure S4B).

### 2.3 Infection with genotoxic *Salmonella* prevents cell cycle arrest in APC‐deficient cells

**Figure 4:**
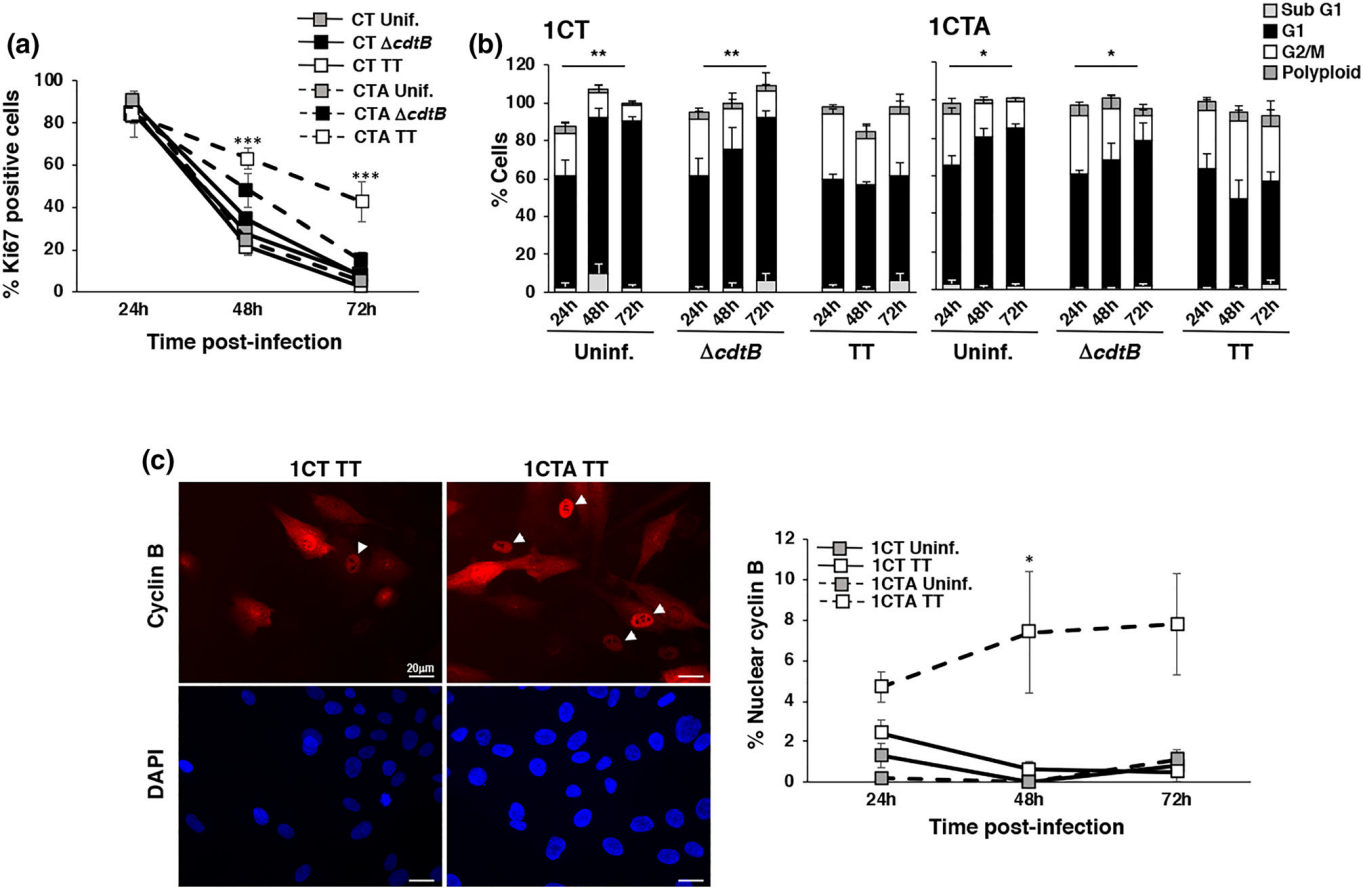
APC deficiency impairs activation of cell cycle arrest. 1CT and 1CTA cells grown in 2D culture were left uninfected or infected with the MC1 ∆*cdtB* or MC1 TT strains as described in Figure 1. The proliferation status was assessed by immunofluorescence analysis, using antibodies specific for Ki67 and cyclin B1. (a) Quantification of cells positive for Ki67. Mean ± SEM of four independent experiments. (b) Cell cycle distribution assessed by PI staining followed by FASC analysis. Mean ± SEM of four independent experiments. Statistical analysis was performed using the Student *t* test *p < .05; **p < .01. Significant accumulation in the G1 phase of the cell cycle was observed in 1CT and 1CTA cells at 72‐hr postinfection compared with the 24‐hr time point. (c) Left panel: fluorescence micrographs showing the subcellular distribution of cyclin B1, white arrowheads indicated cells progressing to the M phase of the cell cycle, characterised by the nuclear translocation of cyclin B1. Right panel: quantification of cells with nuclear translocation of cyclin B1. Mean ± SEM of three independent experiments. One hundred cells were evaluated for each experiment for each cell line. Statistical analysis was performed using the Student *t* test, *p < .05; ***p < .001

**Figure 5:**
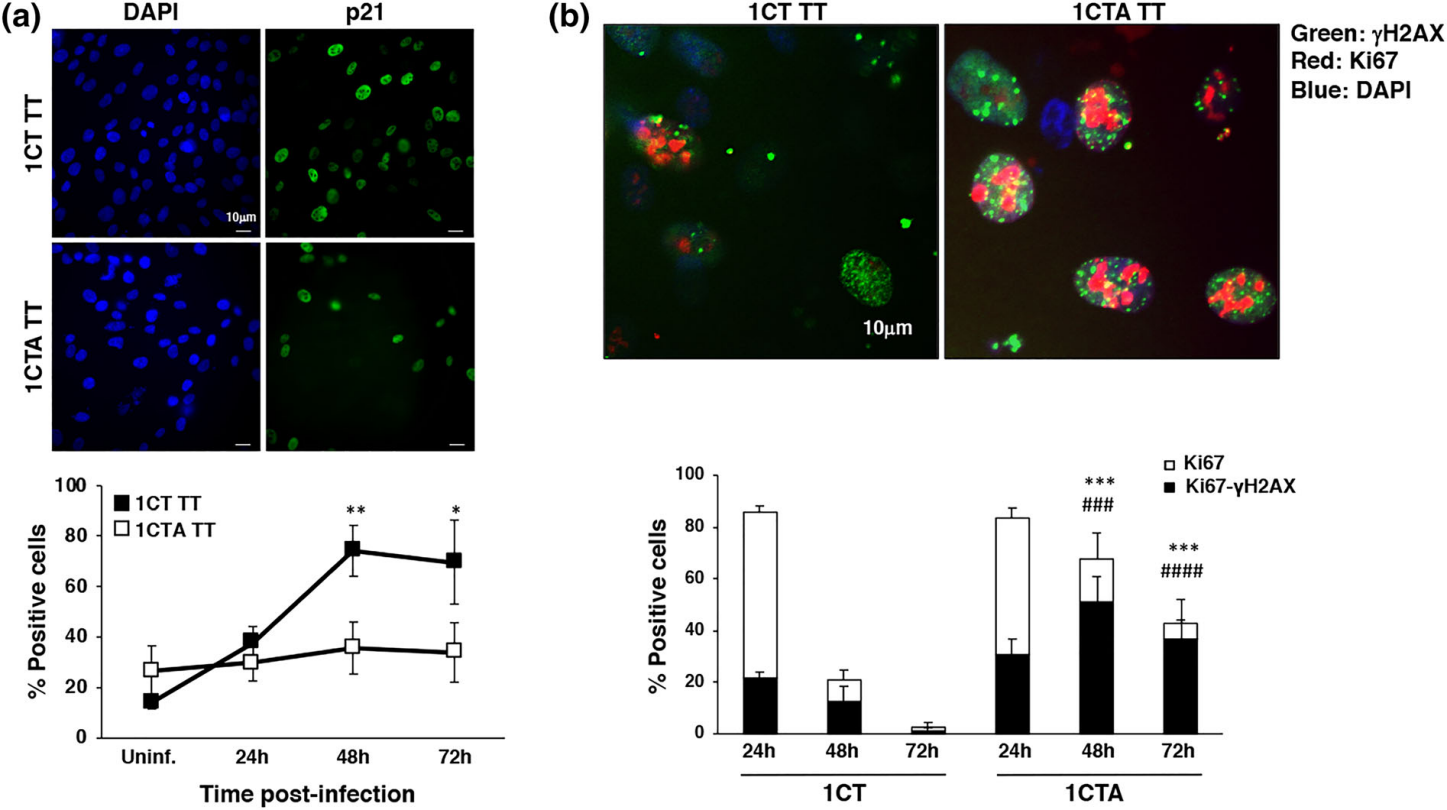
APC deficient cells proliferate in spite of the presence of DNA damage. 1CT and 1CTA cells grown in 2D culture were infected with the MC1 TT strain as described in Figure 1. (a) Upregulation of p21 expression was assessed by immunofluorescence analysis. Upper panel: representative fluorescence micrographs; lower panel: quantification of the positive cells. Mean ± SEM of three to four independent experiments. Statistical analysis comparing 1CT and 1CTA for each time point was performed using the Student *t* test: *p < .05; **p < .01. (b) Induction of proliferation status and DNA damage was assessed by immunofluorescence analysis, using antibodies specific for Ki67 and γH2AX, respectively. Upper panel: representative micrograph showing cells double positive for the proliferation (Ki67, red) and DNA damage (γH2AX, green) markers. Nuclei were counterstained with DAPI (blue). Lower panel: quantification of 1CT and 1CTA cells infected with the MC1 TT strain positive for Ki67 (white bar) and double positive for both Ki67 and γH2AX (black bar). Mean ± SEM of three to four independent experiments. Statistical analysis was performed using the Student *t* test. *^,#^ *p* < .05; **^,##^ *p* < 0.01; ***^,###^ *p* < .001; ****^,####^ *p* < .0001. ^*^comparison Ki67 positive cells, ^#^comparison Ki67‐γH2AX double‐positive cells

To evaluate the effect of the sustained DDR and failure to repair the genotoxin‐induced DNA damage on cell proliferation, we monitored the levels of Ki67 expression and the rate of cell growth in untreated, MC1 TT or MC1 Δ*cdtB* infected 1CT and 1CTA cells. As shown in Figure 4a, in the absence of bacterial infection or upon infection with the control MC1 Δ*cdtB* strain, both APC‐proficient and APC‐deficient cells exhibited contact inhibition over the course of the experiment, as assessed by a time‐dependent decrease in the number of Ki67 positive cells and a significant accumulation of cells in the G1 phase of the cell cycle at 72‐hr postinfection (Figure 4b). The percentage of Ki67 positive cells also decreased in infected 1CT cells following activation of the DDR response, reaching more than 90% of the total population 72‐hr postinfection (Figures 4a). Cell cycle analysis showed that cells were arrested both in G1 and G2 phases of the cell cycle (Figure 4b). In contrast, approximately 50% of the 1CTA cells remained Ki67 positive 72‐hr postinfection with the genotoxic *Salmonella* (Figure 4a), and this effect was not associated with accumulation of cells in the G2 phase of the cell cycle (Figure 4b). Growth curve analysis further showed that although 1CT cells infected with the MC1 TT strain show a significant reduced cell recovery compared with the uninfected cells 72‐hr postinfection, this effect was not observed in infected 1CTA cells, where the cell recovery was similar to that observed in uninfected controls (Figure S3B). The lower cell recovery in the infected 1CT cells was not due to increased rate of cell death, because the percentage of subG1 apoptotic cells was not significantly different in the two cell lines at any condition or time point tested (Figure 4b). Progression from G2 to mitosis in the 1CTA cells exposed to the genotoxic strain was further confirmed by nuclear translocation of cyclin B1 (Pines & Hunter, 1991; Figure 4c) and was associated with failure to upregulate expression of the cyclin‐dependent kinase inhibitor p21 (Figure 5a; Georgakilas, Martin, & Bonner, 2017). It is noteworthy that the majority of the CT1A cells positive for Ki67 at 48‐hr and 72‐hr postinfection still maintained sustained levels of DNA damage, visualised by staining for γH2AX (Figure 5b), indicating failure to properly activate the cell cycle checkpoint response.

### 2.4 APC loss enhances genomic instability upon infection with the genotoxic *Salmonella*

**Figure 6:**
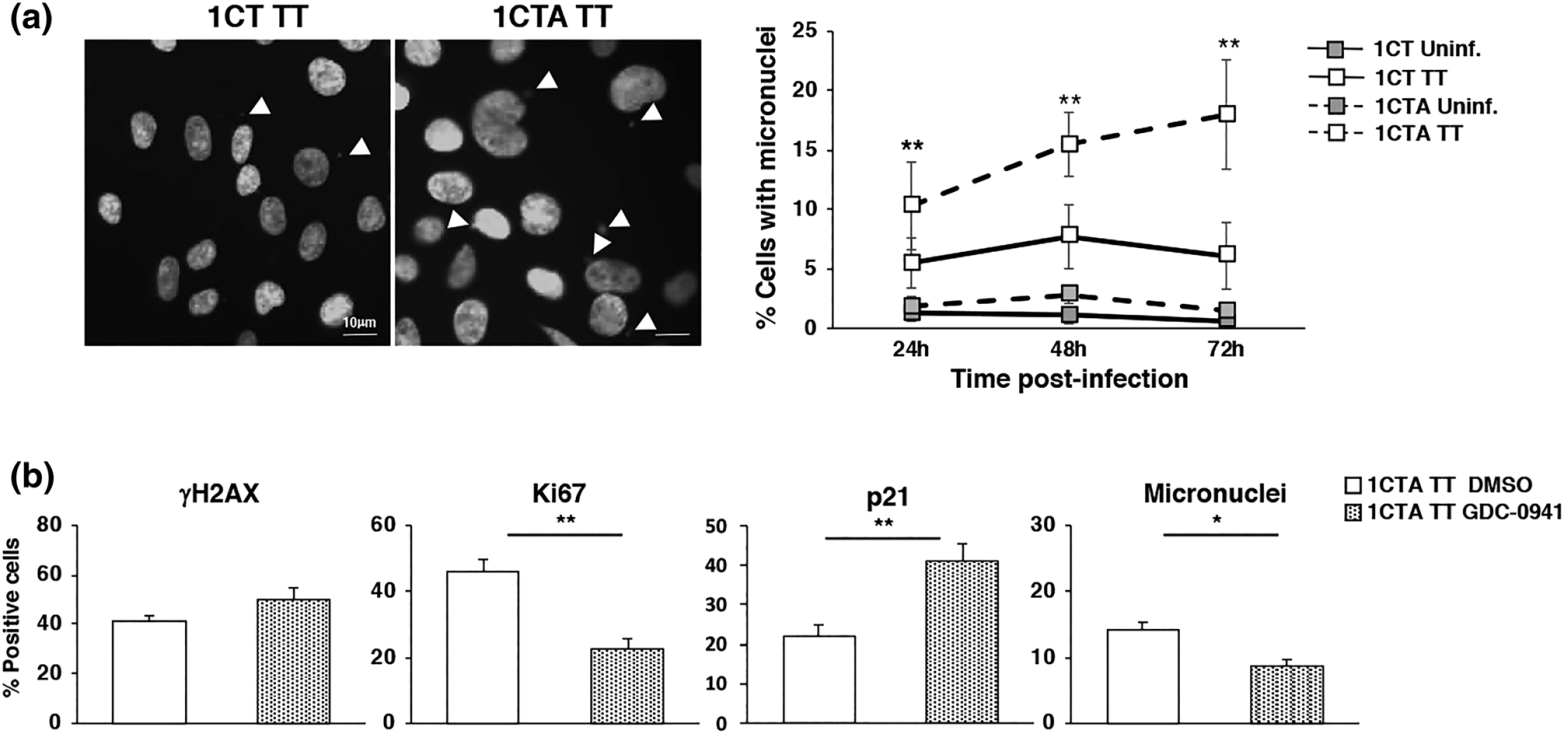
APC deficiency promotes acquisition of genomic instability upon infection with the genotoxic *Salmonella* in a PI3K dependent manner. (a) 1CT and 1CTA cells, grown in 2D culture, were left untreated (Uninf.) or infected with the MC1 TT (TT) strain at MOI 100:1 for the indicated period of time. The left panel shows representative fluorescence micrographs of the infected cells illustrating the presence of micronuclei (white arrowheads) detected by DAPI staining. The right panel shows the quantification of micronuclei positive cells. Mean ± SEM of four to eight independent experiments. (b) 1CTA cells were pretreated for 1 hr with the specific PI3K inhibitor CDG‐0941 (1 μM) for 1‐hr prior infection, which was carried on for 72 hr. DMSO was used as vehicle control. Detection of DNA damage, proliferative capacity, and micronuclei formation were assessed by γH2AX, Ki67, p21, and DAPI staining. Mean ± SEM of three independent experiments. Statistical analysis was performed using the Student *t* test, *p < .05; **p < .01

We next assess whether the reduced DNA repair capacity combined with the failure to halt cell proliferation observed in APC‐deficient cells exposed to the genotoxic *Salmonella* was associated with enhanced genomic instability. To this end, 1CT and 1CTA cells were left uninfected, or infected with MC1 TT, or control MC1 Δ*cdtB* bacteria (data not shown), and genomic instability was assessed by the formation of micronuclei over a period of 72 hr. As shown in Figure 6a, a marginal increase of micronuclei positive cells was detected in the 1CT cells infected with the MC1 TT strain, consistent with the fact that the majority of the cells stop proliferation in response to infection with the genotoxin‐producing bacterium (Figure 4). In contrast, a significant higher percentage of cells carrying micronuclei was observed 24‐hr postinfection in 1CTA cells exposed to the genotoxic *Salmonella*, and the rate of micronuclei positive cells increased over time. Similar time‐dependent increase in the formation of micronuclei was observed in infected murine *Apc* ^*+/Min*^ cells (Figure S4C). We did not detect a significant increase in the percentage of polyploid cells over the course of 72‐hr infection with the MC1 TT strain (Figure 4b).

*Salmonella* infection induces activation of the PI3K/AKT axis and the mitogen‐activated protein kinase (MAPK) p38 (Hobbie, Chen, Davis, & Galan, 1997; Steele‐Mortimer et al., 2000), which have also been demonstrated to activate survival and proliferative signals critical in carcinogenesis (Dolado & Nebreda, 2008; Liu, Cheng, Roberts, & Zhao, 2009). To assess whether these pathways contribute to inhibit the checkpoint response upon MC1 TT infection in APC‐deficient cells, leading to accumulation of genomic instability, 1CTA cells were preincubated with the inhibitors GDC‐0941 (1 μM) and SB203580 (10 μM) specific for PI3K and MAPK p38, respectively, prior infection. Treatment did not affect infection (data not shown) or the extent of the *Salmonella*‐induced DNA damage 72‐hr postinfection as assessed by the similar levels of phosphorylation of γH2AX (Figure 6b). However, inhibition of the PI3K, but not MAPK p38, reduced the number of cells positive for the proliferative marker Ki67 in cells infected with the genotoxic *Salmonella* (Figure 6b, and data not shown) and cell outgrowth as assessed by colony assay in cells exposed to purified CDT (Figure S6). The reduced proliferative activity observed in 1CTA treated with the PI3K inhibitor was dependent on the upregulation of p21 expression (Figure 6b). Collectively, these data indicate that inhibition of the checkpoint responses is, at least partially, dependent on the PI3K/AKT axis. As a consequence of the reduced proliferative state, PI3K inhibition resulted in reduction of the percentage of cells carrying micronuclei (Figure 6b). We confirmed that the levels of Ki67 expression and micronuclei formation were significantly lower in 1CT cells infected with the toxigenic strain compared with the APC‐deficient cells, and this phenotype was not affected by the inhibitors (data not shown).

### 2.5 Sustained activation of the DDR was confirmed in an organotypic 3D‐tissue model of colonic epithelium

Because the 2D monoculture system does not reproduce the microenvironment of the infected epithelium and its interaction with the connective tissue of the lamina propria, we developed a 3D organotypic culture, where primary colonic fibroblasts embedded into a collagen matrix provided a lamina propria‐like support for the colonic epithelial cell monolayer (Figure S7A). We observed that epithelial cells grown in 3D conditions were more susceptible to *Salmonella* infection, as illustrated by bacterial overgrowth and destruction of the epithelial monolayer already 24‐hr postinfection at MOI 100:1 and 50:1 (Figure S7B). The infection of 1CT and 1CTA cells with MC1 TT or MC1 Δ*cdtB* bacteria at MOI 25:1 resulted in the formation of *Salmonella*‐containing vacuoles, detected by LPS staining 24‐hr postinfection (Figure S7C), without major alteration of the epithelial layer and was therefore used in all the subsequent experiments.

**Figure 7:**
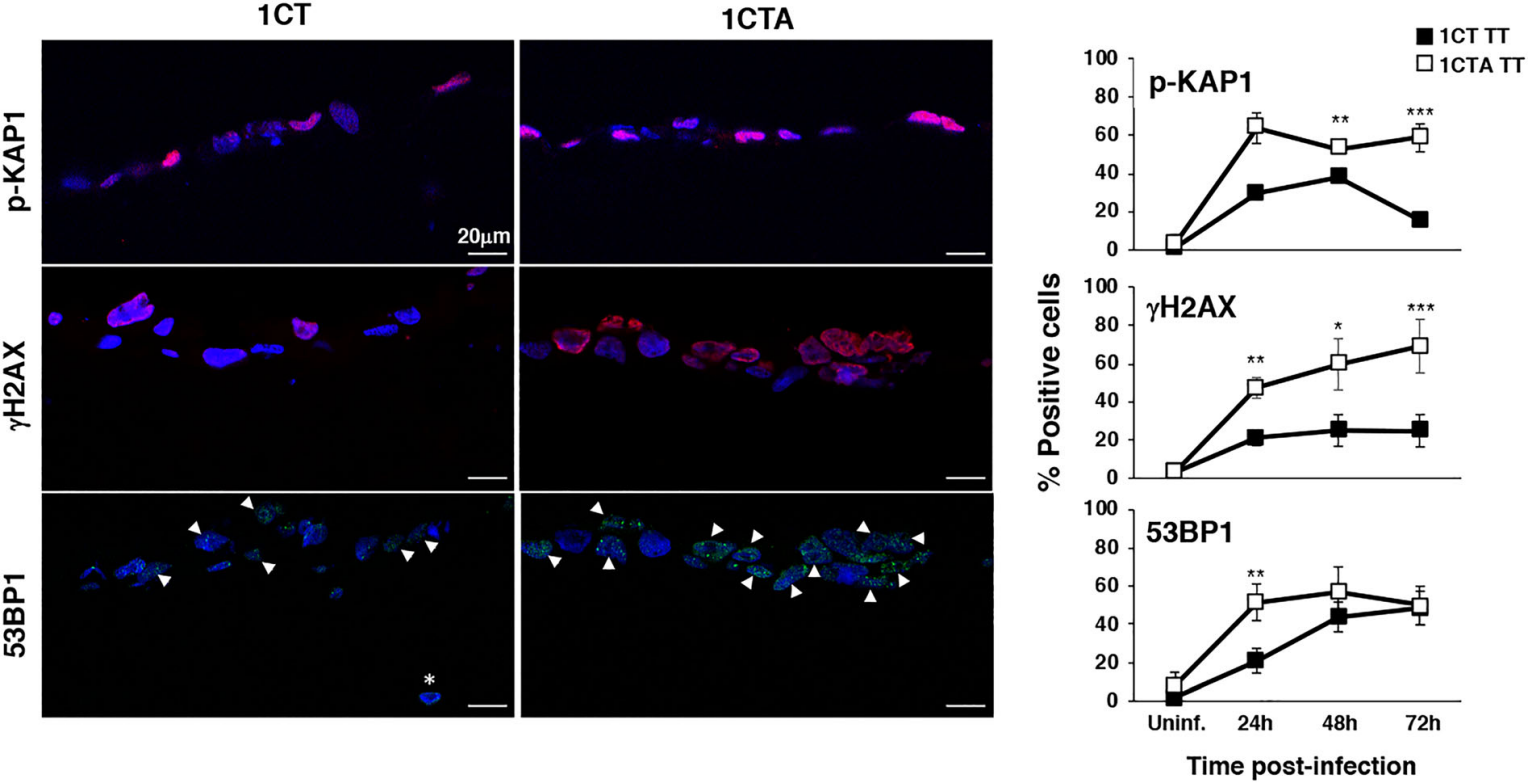
APC‐deficient cells show a sustained activation of the DNA damage response in 3D culture. 1CT and 1CTA cells, grown in 3D culture, were left untreated (Uninf.) or infected with the MC1TT strain (TT) at MOI 25:1 for the indicated period of time. Activation of the DNA damage response was assessed as described in Figure 3. Left panel: representative scanning confocal micrographs; right panel: quantification of the positive cells. Mean ± SD of three to seven independent experiments. One hundred cells were evaluated for each experiment for each cell line. Statistical analysis was performed using the Student *t* test, *p < .05; **p < .01; ***p < .001

**Figure 8:**
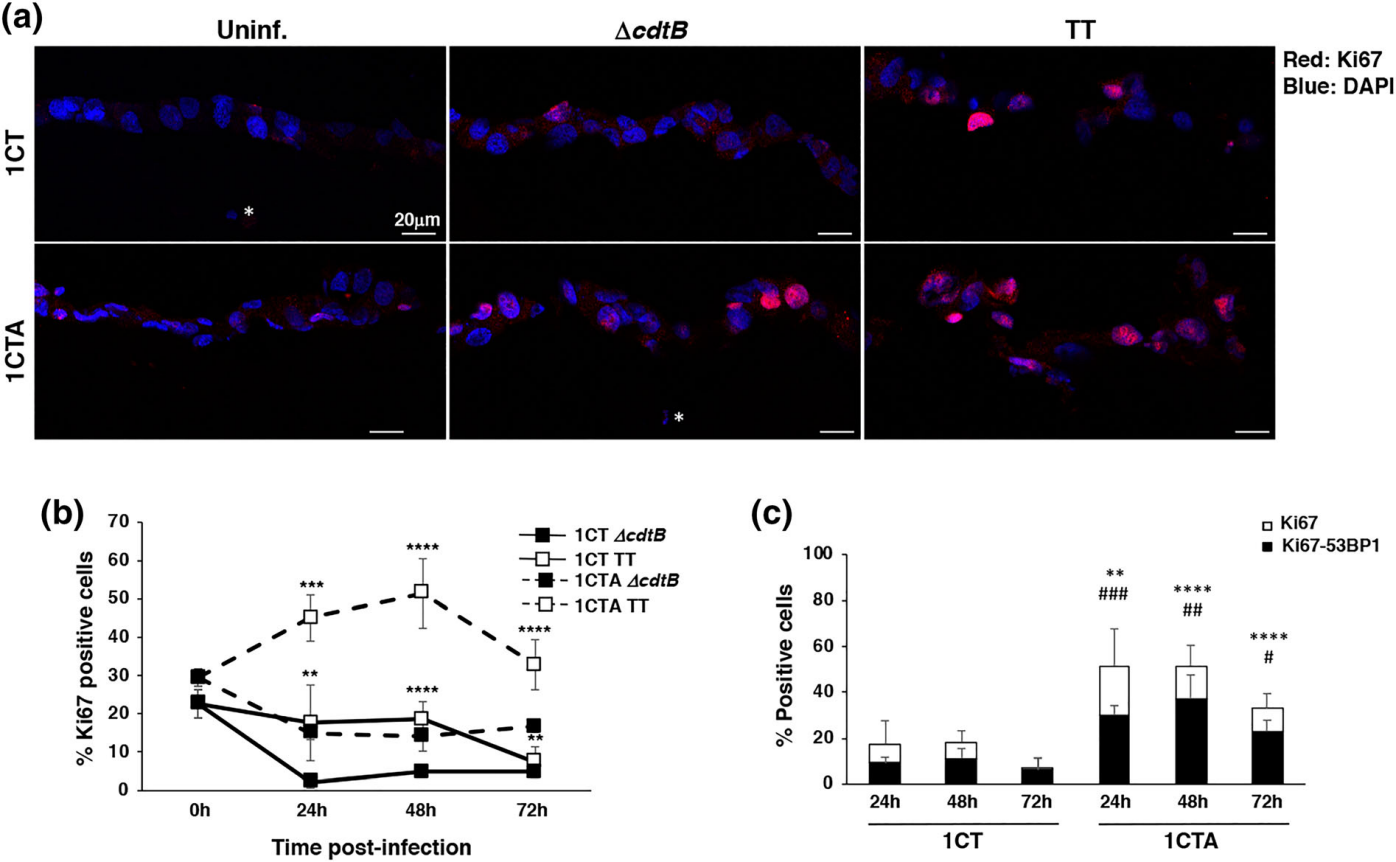
Infection with genotoxic *Salmonella* promotes exit from quiescence of the 1CTA cells. 1CT and 1CTA cells, grown in 3D culture, were infected with the MC1 TT or MC1 ∆*cdtB* strain. Induction of proliferation status and DNA damage was assessed by immunofluorescence analysis, using antibodies specific for Ki67 and 53BP1, respectively, at the indicated time points. (a) Representative scanning confocal micrograph of infected 1CT and 1CTA cells grown in 3D culture stained with the anti‐Ki67 specific antibody (red). Nuclei were counterstained with DAPI (blue). Magnification 40×. The white asterisk indicates nuclei of fibroblasts embedded in the collagen matrix. (b) Quantification of cells positive for Ki67. (c) Quantification of cells positive for Ki67 (white bar) and double positive for both Ki67 and 53BP1 foci (black bar). Mean ± SEM of three to four independent experiments. Statistical analysis was performed using the Student *t* test. *^,#^ *p* < .05; **^,##^ *p* < .01; ***^,###^ *p* < .001; ****^,####^ *p* < .0001. ^*^comparison Ki67 positive cells, ^#^comparison Ki67‐γH2AX double‐positive cells

The 3D model confirmed the sustained activation of the DDR in infected APC‐deficient 1CTA cells compared with the 1CT cell line (Figure 7). However, whereas the majority of 1CT and 1CTA cells grown in 2D culture expressed the Ki67 proliferation marker at the initial stages of the experimental set‐up (Figure 4a), the 3D culture condition promoted a significant reduction in the percentage of Ki67 positive cells from approximately 80% to 25% in uninfected controls (cf. Figures 4a and 8b) due to formation of a confluent monolayer (Figure S7A). As expected, infection with MC1 TT induced a further decrease in the percentage of Ki67 positive cells in 1CT monolayers. In contrast, the percentage of 1CTA cells expressing the proliferative marker increased up to 50% 24‐hr postinfection and remained high for the entire observation period (Figure 8b), suggesting an exit from the quiescent state upon MC1 TT infection. This effect was specific for the genotoxic *Salmonella*, because it was not observed in cells infected with the MC1 ∆*cdtB* strain (Figures 8b). In addition, the majority of Ki67 positive cells showed signs of DNA damage, assessed by formation of the 53BP1 foci (Figure 8c).

## 3 DISCUSSION

The microbiota of CRC patients has been shown to harbour an increased proportion of bacteria expressing bacterial genotoxins (Buc et al., 2013; Candela et al., 2011; Candela et al., 2014; Kostic, Xavier, & Gevers, 2014; Martin et al., 2004; Shen et al., 2010). The protumorigenic effect of these genotoxins has been shown in mouse models prone to develop cancer or dysplasia, such as the IL10 knockout mice exposed to the procarcinogen AOM (Arthur et al., 2012). However, the context in which the infection/colonisation with genotoxin‐producing bacteria occurs may have a great impact on the outcome of the response to the toxins. Indeed, it has been shown that infection of immunocompetent mice with a TT producing *Salmonella* is associated with a strong suppression of the intestinal inflammatory response, and no sign of dysplasia or tumour development was observed in mice colonised with the bacterium for 6 months (Del Bel Belluz et al., 2016). Thus, the procarcinogenic effect of these toxin families may be exerted under special circumstances, such as a proinflammatory environment or haploinsufficiency of tumour suppressor genes.

In this study, we have demonstrated that the lack of the *APC* tumour suppressor gene, mutated in 80% of sporadic CRC and in 100% of FAP (reviewed in Armaghany, Wilson, Chu, & Mills, 2012), contributed to the carcinogenic properties of the typhoid toxin, in colonic epithelial cells using both classical monolayer 2D culture and 3D organotypic model, by inhibiting concomitantly two key steps of the DDR: DNA repair and DNA damage‐induced cell cycle arrest. We showed that in response to infection with the TT‐producing *Salmonella*, APC‐deficient or haploinsufficient cells present (a) a sustained DDR response (Figures 2, 7, and S4), (b) a reduced capacity to repair DNA damage in response to a broad panel of genotoxic agents (Figures 3 and S4), and (c) a failure to induce upregulation of the cyclin kinase inhibitor p21 and consequently to activate a proper cell cycle arrest leading to proliferation in spite of the presence of DNA damage (Figures 4, 5, and 8). The inability to repair DNA and to completely halt the cell cycle is ultimately associated with an enhanced acquisition of genomic instability (Figures 6 and S4), thus synergising with the APC deficiency in the carcinogenic process. The failure to activate a proper checkpoint response was dependent on activation of the PI3K pathway, as shown by the significant reduction in cells expressing the proliferation marker Ki67 and consequent reduction in micronuclei formation in cells pretreated with the specific PI3K inhibitor (Figure 6). *Salmonella* infection is associated with the activation of the PI3K/AKT axis (Steele‐Mortimer et al., 2000); however, in our experimental set‐up, the ability to inhibit the checkpoint responses and maintain Ki67 expression was predominantly observed in cells infected with the genotoxic strain compared with the effect induced by the control strain MC1 Δ*cdtB* (Figures 4a and 8b), which is consistent with activation of PI3K and AKT observed in response to CDT or ionising radiation (Frisan, Cortes‐Bratti, Chaves‐Olarte, Stenerlow, & Thelestam, 2003; Seiwert et al., 2017). The activation of the PI3K pathway is most likely dependent on the activation of the DDR, because it has been previously demonstrated that Nbs1, one of the three components of the Mre11‐Rad50‐Nbs1 complex, interacts through its conserved C‐terminal motif with the p110α catalytic subunit of PI3K, stimulating the kinase activity (Chen et al., 2008), and we have previously shown the activation of this complex in cells exposed to CDT (Guerra et al., 2010; Li et al., 2002). However, in APC‐deficient cells, this activation can be further potentiated because CKS2, a direct transcriptional target of the β catenin/TCF4, has been shown to activate the PI3K pathway in CRC cells (Qi et al., 2016). As a consequence of the PI3K/AKT pathway activation, downregulation of p21, likely mediated by the AKT‐dependent inhibition of the FoxO transcription family (Zhang, Tang, Hadden, & Rishi, 2011), overrides the checkpoint response, because cells exposed to the PI3K inhibitor showed reduced Ki67 levels and concomitant upregulation of p21 expression (Figure 6b). In addition, colonisation with the CDT producing *Campylobacter jejuni* has been shown to enhance tumour formation in the *Apc* ^*Min/+*^ mouse model upon dextran sulfate sodium‐induced colitis, in a mechanistic target of rapamycin‐dependent manner (mTOR) (He et al., 2019). Interestingly, mTOR is a key target of the PI3K/AKT axis (Fruman et al., 2017), highlighting the relevance of the activation of this pathway in bacterial genotoxin‐induced carcinogenesis in the context of APC deficiency.

It is noteworthy that a small but reproducible fraction of the APC‐proficient 1CT cells grown in 3D culture presented a sign of DNA damage but were still positive for the proliferation marker Ki67 (Figure 8). Thus, it is possible that under certain circumstances, also normal cells may escape the checkpoint response, which can lead to acquisition of genomic instability and transformation. These circumstances may be a chronic inflammatory condition or coinfection with other bacteria known to be associated with CRC, such as *Fusobacterium nucleatum* (Kostic et al., 2012) and the enterotoxigenic *Bacteroides fragilis* (Purcell et al., 2017). Interestingly, recent data showed that the colonic mucosa of FAP patients is characterised by the presence of bacterial biofilms, where *E. coli* and *B. fragilis* are predominant members and that the two bacteria synergised to promote cancer in an AOM‐dependent CRC murine model (Dejea et al., 2018).

Collectively, these data highlight the carcinogenic potential of the TT and provide a molecular mechanism to support the epidemiological observations linking chronic *Salmonella* infection with increased cancer risk not only in the hepatobiliary tract but also in the colon (Dutta et al., 2000; Mughini‐Gras et al., 2018).

### 3.1 APC deficiency and DNA damage repair

Our data, demonstrating an altered DNA repair in the APC‐deficient cells, are in agreement with previous results demonstrating that intoxication with sublethal doses of the *E. coli* CDT synergised with the APC deficiency in promoting genomic instability (Graillot et al., 2016). We expanded this analysis by performing a detailed analysis of the DDR and further showed that APC deficiency is associated with failure to repair not only DNA single‐ and double‐strand breaks but also oxidative damage, indicating that APC is involved in the regulation of multiple repairs pathways. This protein is well‐characterised as negative regulator of β‐catenin. The absence of a functional APC results in a constitutive translocation of β‐catenin in the nucleus leading to transcriptional activation of the target genes, many of which promote cell cycle division (cMYC and Cyclin D1; reviewed in Tortelote, Reis, de Almeida Mendes, & Abreu, 2017). However, there are evidences that APC can directly regulate DNA repair in a β‐catenin independent manner. Kouzmenko and colleagues have shown that APC (aminoacids 1441‐2077) contributes to the recruitment of DNA‐PK, a key sensor and regulator of the non‐homologous end joining repair and favours phosphorylation of histone H2AX (γH2AX) in response to DNA‐damaging agents causing DNA DSB (Kouzmenko, Takeyama, Kawasaki, Akiyama, & Kato, 2008; Wang & Lippard, 2005). Interestingly, germline and somatic *APC* mutations resulting in a truncated form of the protein are localised within this region (Kohler, Derungs, Daum, Behrens, & Schneikert, 2008). This effect seems to be specific for certain types of DNA damage, such as induction of DNA breaks, whereas APC does not interact with γH2AX in cells exposed to cisplatin, which promotes the formation of platinum‐DNA adducts, mainly repaired by nucleotide‐ and base‐excision repair (Kouzmenko et al., 2008; Wang & Lippard, 2005). Alternatively, failure to repair the damaged DNA may be dependent on the lack of p21 upregulation observed in APC‐deficient cells (Figure 5a), which has been shown to accumulate at the sites of laser‐induced localised DNA breaks, colocalising with γH2AX and Ku80, one of the components of the DNA‐PK (Koike, Yutoku, & Koike, 2011).

### 3.2 Genotoxin‐producing bacteria and CRC

It is not clear whether the dysbiosis observed in CRC patients precedes the tumour development, contributing to the initiation, or is a consequence of the altered microenvironment, contributing to tumour progression. It has been shown that the microbiota of the APC^Min∆716/+^ and APC^Min∆850/+^ mice, widely used models for FAP, displays an enrichment in *E. coli* and *B. fragilis* compared with the wild‐type control mice even prior the development of polyposis (Dejea et al., 2018; Son et al., 2015), indicating that the tumour suppressor gene mutation causes the dysbiosis.

Two major forms of genomic instability have been detected in CRC: chromosomal instability (CIN), associated with a poor prognosis and characterised by frequent deletions, amplifications, inversions, and translocations; and microsatellite instability, with a more favourable prognosis and characterised by a wide spread hypermethylation at promoter sites (reviewed in Amaro, Chiara, & Pfeffer, 2016). CIN occurs more frequently in tumour showing APC and KRAS mutations, whereas the microsatellite instability phenotype is mainly associated with BRAF mutations (reviewed in Amaro et al., 2016). Chronic exposure to sublethal dose of the *Helicobacter hepaticus* CDT induces genomic instability in rat fibroblasts, and approximately 20% of the alterations observed were deletions (Guidi, Guerra, et al., 2013), suggesting that the presence of genotoxin‐producing bacteria can contribute to the CIN observed in a subset of CRC patients.

### 3.3 Organotypic culture

In this study, we have compared the effects of the infection with the genotoxic *Salmonella* in classical 2D conditions and in 3D organotypic model. The two models showed a similar alteration of the DDR kinetics in APC‐deficient cells (Figures 2 and 7). However, differently from the 2D model where both 1CT and 1CTA cells were replicating as indicated by the high percentage of Ki67 positive cells in control and infected cells at the 24‐hr time point, cells grown in 3D were approaching a quiescent stage when infection was performed (Figure 8). Interestingly, infection with the toxigenic strain promoted an increase in the percentage of 1CTA cells positive for Ki67 in the 3D model. This outcome was not detected in cells infected with the control MC1 ∆*cdtB Salmonella* (Figure 8), excluding that the cell proliferation is due to a wound‐healing response to the infection. We are currently analysing the cause(s) of this different behaviour; however, the data strongly suggest that more complex culture settings may highlight aspects of the cellular responses to DNA damage that were previously not observed in traditional monocellular systems.

In conclusion, we demonstrate that infection with genotoxin‐producing enteric bacteria, enriched in the microbiome of CRC patients, synergises with the APC deficiency in the acquisition of genomic instability, the enabling characteristic of cancer, due to an altered DNA repair, activation of PI3K, and reduced capacity to implement an efficient cell cycle arrest. In addition, the use of organotypic 3D culture may represent a more suitable model than the classical 2D conditions to identify still unknown outcomes of the cell response to genotoxic stresses.

## 4 EXPERIMENTAL PROCEDURES

### 4.1 Cell lines

The isogenic human colonic epithelial cell lines 1CT (APC wild type) and 1CTA (APC deficient) were previously described (Roig et al., 2010). Cells were cultured in a high‐glucose Dulbecco's Modified Eagle (DMEM) medium/medium 199 (Sigma‐Aldrich Merck, Darmstadt, Germany, at ratio 4:1), supplemented with 2% fetal bovine serum (FBS; Gibco ThermoFisher Scientific), epidermal growth factor (EGF 20 ng/ml, Sigma‐Aldrich), hydrocortisone (1 mg/ml, Sigma‐Aldrich), insulin (10 mg/ml, Sigma‐Aldrich), transferrin (2 mg/ml, Sigma‐Aldrich Merck), sodium selenite (5 nM, Sigma‐Aldrich Merck), and gentamycin sulfate (50 μg/ml, Sigma‐Aldrich Merck): DMEM complete medium. The 1CTA cells were cultured in DMEM complete medium supplemented with puromycin (1 μg/ml, Invitrogen, Carlsbad, CA, USA).

Human colonic fibroblasts (ATCC, Manassas, VA, USA) were maintained in Eagle's Minimum Essential Medium (ATCC) supplemented with 10% of FBS (Gibco) and 10 μg/ml of ciprofloxacin (Sigma‐Aldrich Merck).

The immortalised isogenic murine *Apc* ^*+/+*^ and *Apc* ^*+/Min*^ colon epithelial cells were previously described (Forest et al., 2003). The cell lines harbour a temperature‐sensitive mutation of the simian virus 40 large tumour antigen gene (tsA58), under the control of interferon‐γ (IFN‐γ) and express an active SV40 form at the permissive temperature (33°C). Cells were routinely cultured at permissive temperature of 33°C in DMEM (Sigma‐Aldrich Merck) supplemented with 10% FBS (Gibco), EGF (10 ng/ml, Sigma‐Aldrich), IFN‐γ (2 ng/ml, BD Biosciences, Franklin Lakes, NJ, USA), and 2% penicillin/streptomycin (ThermoFisher Scientific).

All cells were maintained in a humidified atmosphere with 5% CO_2_ at 37°C.

When indicated, cells were preincubated for 1 hr with inhibitors specific for the MAPK p38 (SB203580 10 μM, Sigma‐Aldrich Merck) or PI3K (GDC‐0941 1 μM, Abcam, Cambridge, UK) or DMSO (Sigma‐Aldrich Merck) as vehicle, prior infection. Infection was performed in in the continuous presence of SB203580, whereas 1‐hr treatment with the GDC‐0941 was repeated every 24 hr after infection.

### 4.2 RT‐qPCR

RNA was isolated using the RNeasy Mini kit (Qiagen, Hilden, Germany), and cDNA synthesis was performed using the High‐capacity cDNA Reverse Transcription and RiboLock RNase Inhibitor kits (Thermofisher Scientific) with 1‐μg RNA and a PTC‐225 Thermal Cycler (MJ Research, Waltham, MA, USA), according to the instructions of the manufacturer. Quantitative PCR was performed using the following primers F: AGGCTGCATGAGAGCACTTGTG and R: CACACTTCCAACTTCTCGCAACG for *APC* and F: CTGGCACCCAGCACAATG and R: GCCGATCCACACGGAGTACTT for beta actin. Amplifications were carried out using an ABI PRISM® 7000 Sequence Detection System (Applied Biosystems, Foster City, CA, USA) as follows: a first one‐hold stage at 95°C for 10 min followed by 41 cycles (95°C for 15 s, 60°C for 30 s, and 72°C for 30 s), and a final extending step (95°C for 10 s). All qPCR reactions were performed in triplicate, and the threshold cycle (Ct) values were averaged. The fold change of the target gene relative to the internal control was calculated as 2^−Δ(ΔCt)^_,_ where ΔCt = Ct_target_ – Ct_housekeeping_ and Δ (ΔCT) = ΔCt_treated_ – ΔCt_untreated_, according to the Minimum Information for Publication of Quantitative Real‐Time PCR Experiments guidelines.

### 4.3 Organotypic 3D tissue models

Colonic tissue models were prepared based on a protocol for lung tissue model previously described (Mairpady Shambat et al., 2015; Sundstrom et al., 2016), with minor modifications. Briefly, a six‐well plate insert with 3.0‐μm high‐density membrane (VWR, Radnor, PA, USA) was coated with 1 ml of a solution of bovine type I collagen (PureCol, Cell systems, Kirkland, WA, USA) in DMEM at a final concentration of 0.72 mg/ml (collagen‐DMEM) and incubated for 30 min at 37°C in 5% CO_2_. Eighty thousand human colonic fibroblasts were resuspended in 3 ml of the collagen–DMEM solution, overlaid on the polymerised collagen layer and incubated for 2 hr at 37°C in 5% CO_2_. Following the polymerisation, a 2‐ml DMEM medium was added to the outer chamber, and the culture was kept for 24 hr at 37°C in 5% CO_2_. The subsequent day, 2 ml of fresh DMEM medium were added to the inner chamber. The models were maintained for 7 days, and the medium was replaced every second day. Six hundred thousand 1CT or 1CTA cells, resuspended in 150‐μl DMEM complete medium without puromycin and gentamycin, were overlaid on the fibroblast/collagen matrix and incubated for 4 hr at 37°C in 5% CO_2_ after which 2‐ml complete DMEM medium without antibiotics was gently added to the insert, and the models were cultivated for additional 24 hr at 37°C in 5% CO_2_ prior infection.

### 4.4 Bacterial strains

The *Salmonella typhimurium* strain MC1 used in this study was previously described (Clements et al., 2002). The strain was transformed with the pEGFP‐C1 plasmid carrying a cassette containing the TT genes *pltB‐pltA* and *cdtB* (MC1 TT). As control, the MC1 strain was transformed with the pEGFP‐C1 vector containing only the *pltB‐pltA* genes (MC1 *ΔcdtB*). Cloning of the TT genes into the pEGFP‐C1 plasmid was previously described (Guidi, Levi, et al., 2013).

### 4.5 Bacterial infection

Fifty thousand 1CT and 1CTA or sixty thousand *Apc* ^*+/+*^ and *Apc* ^*+/Min*^ cells were grown in six well plates (Primaria^TM^, Gibco ThermoFisher Scientific), without or with 13‐mm‐diameter coverslips previously coated with FBS, in 3‐ml complete medium.

Epithelial cells were infected at an MOI of 100:1 in a gentamicin protection assay as previously described (Bjur, Eriksson‐Ygberg, Aslund, & Rhen, 2006). Cells were lysed in 1 ml of 0.5% s*odium deoxycholate* (Sigma‐Aldrich) in PBS to evaluate bacterial load by CFU counting as previously described (Bjur et al., 2006). Cells grown on slides were fixed in 4% formaldehyde in phosphate‐buffered saline (PBS) for 20 min at room temperature for immunofluorescence analysis.

Human organotypic colonic models were infected at an MOI of 25:1 as previously described (Bjur et al., 2006), except that the models were not subjected to centrifugation, and incubation with the bacterial suspension was prolonged to 2 hr. After infection, cells were maintained in RPMI supplemented with 10% FBS and 10‐μg/ml gentamicin for the indicated periods of time, when 2 ml of 2‐M sucrose solution were added to both the inner and the outer chambers, and samples were incubated for 1 hr at room temperature. Subsequently, the models were embedded in O.C.T. (Sakura Finetek, Torrance, CA, USA), snap frozen and stored at −80°C. Six‐μm sections were cut using a CryoStar™ NX70 Cryostat (Gibco ThermoFisher Scientific), and slides were fixed in 4% PFA in PBS for 20 min at room temperature for immunofluorescence analysis.

### 4.6 Repair assay

The capacity of the 1CT/1CTA and *Apc* ^*+/+*^/*Apc* ^*+/Min*^ cells to repair the DNA damage was assessed as previously described with minor modifications (Graillot et al., 2016). Briefly, 100,000 cells were grown on 13‐mm‐diameter coverslips in six well plates in 3‐ml complete medium and treated with the indicated DNA‐damaging agents. After 6 hr, cells were either fixed in 4% formaldehyde in PBS (defined as 6 hr) to assess the extent of the DNA damage or washed once with PBS and incubated with fresh medium in absence of the genotoxic agent for additional 18 hr before fixation (defined as 24 hr) to assess the residual DNA damage. DNA damage was assessed by immunofluorescence analysis as described below. The percentage of repair was calculated as follows: [1 − (% positive cells at 24 hr/% positive cells at 6 hr)] × 100, where cells with a defective repair will accumulate more DNA damage 24‐hr posttreatment, resulting in a lower repair percentage.

The following genotoxic agents were used: the recombinant CDT from *Haemophilus ducreyi* (Guerra et al., 2005; 1 μg/ml), etoposide (15 μM, Sigma‐Aldrich Merck), camptothecin (5 μM, Selleckchem, Munich, Germany), and H_2_O_2_ (50 μM, Sigma‐Aldrich‐Merck).

### 4.7 Immunofluorescence

Immunofluorescence was performed as previously described (Guerra et al., 2008). The following primary antibodies were used: rabbit antibodies anti‐*S*. enterica OMA (1:500, Reagensia AB, Solna, Sweden), anti‐phospho‐KAP1 (1:100, Bethyl Laboratories Inc, TX, USA), anti‐phospho‐H2AX Ser139 (γH2AX), anti‐phospho‐CHK2 Thr68, and anti‐Ki67 (1:100, Cell Signaling Technology, Boston, MA, USA); and mouse antibodies anti‐γH2AX (1:100, Millipore), anti‐53BP1 (1:100, BD), anti‐phospho‐p53 Ser15 (1:100, Cell Signaling Technology), anti‐cyclin B1 (1:100, Santa Cruz Biotechnology, Dallas, TX, USA), and anti‐p21 (Cip1/WAF1; 1:100, BD). The actin cytoskeleton was visualised by staining with TRITC or FITC‐conjugated phalloidin (1 μg/ml, Sigma‐Aldrich Merck) as previously described (Frisan et al., 2003).

Images were acquired with a fluorescence microscope (Leica DM RA2, Leica Microsystems, Wetzlar, Germany) equipped with a CCD camera (C4742‐95, Hamamatsu, Japan) or a confocal scanning microscope (Zeiss Confocal Microscope LSM510 META, Carl Zeiss Microscopy, Jena, Germany). The images were analysed using the ImageJ software, and at least 100 cells per slide were counted. Cells were scored positive for γH2AX and 53BP1 when they exhibited ≥5 foci/cell. Cells were considered positive for phospho‐Kap1, phospho‐Chk2, phospho‐p53, and Ki67 when the mean fluorescence intensity was higher than the mean fluorescence intensity of the uninfected cells plus two standard deviations.

### 4.8 Cell cycle analysis

Cells, harvested by trypsinization, were fixed for 20 min on ice with 1 ml of ice‐cold 70% ethanol and subsequently resuspended in 0.3 ml of propidium iodide solution (3.8‐mM sodium citrate, 0.3% NP40, 0.05‐mg/ml propidium iodide; 0.02 mg/ml RNase) for 1 hr at 4°C. Flow cytometry analysis was performed using a FACSCalibur flow cytometer (BD Biosciences). Pulse processing was used to exclude cell doublets from the analysis. Data from 1 × 10^4^ cells were collected and analysed using the CellQuest Pro software (BD Biosciences).

### 4.9 Colony assay

1CTA cells were preincubated for 1 hr with the specific PI3K inhibitor (GDC‐0941 1 μM), or DMSO as vehicle, prior infection, and exposed to purified CDT at 20 and 10 ng/ml for 2 weeks, 1‐hr treatment with the GDC‐0941 was repeated every 24 hr after infection for 1 week. Cells were fixed with 4% formaldehyde for 20 min, washed twice with 20% methanol, stained with 0.05% crystal violet (Sigma‐Aldrich) in 25% methanol, and washed three times with PBS. Bound stain was extracted by incubation for 15 min in 2% SDS in water and quantified by reading the absorbance at 550 nm.

### 4.10 Statistical analyses

Results are expressed as mean ± SEM of at least three independent experiments. All the statistical analyses have been performed using the Prism 7, Graphpad Software. The significance of differences between two experimental groups was determined by a Student *t* test. The significance of differences between three experimental groups was determined by ANOVA with Fisher's LSD posttest. *P* values <.05 were considered significant.

## CONFLICT OF INTEREST

The authors declare no conflict of interest

## AUTHOR CONTRIBUTIONS

OCBM conceived and carried out the experimental design and wrote the manuscript. AB and FA carried out the experimental design. JWS and JD contributed to the interpretation of the results and to the final version of the manuscript. PC and MS provided experimental support for the 3D organotypic culture and contributed to the final version of the manuscript. MGM conceived the experimental design and provided financial support. TF conceived the experimental design, supervised the project, wrote the manuscript, and provided financial support.

## Supporting information

Figure S1. Phenotypic characterization of APC deficient and proficient cells.
**A.** Representative scanning confocal micrographs showing the staining with TRITC (red)‐ or FITC (green)‐conjugated phalloidin to visualize the actin stress fibers. Nuclei were counterstained with DAPI (blue). Magnification 63X. B. **B.** Nuclear size was assessed using the ImageJ software from micrograph pictures showed in A. Mean ± SEM of three independent experiments. One hundred cells were evaluated for each experiment for each cell line. Statistical analysis was performed using the Student *t* test, ** p < 0.01; *** p < 0.001; **** p < 0.0001.
Figure S2. Infection with the genotoxic *Salmonella* strain induces activation of the DDR
1CT and 1CTA cells, grown in 2D culture, were left uninfected (Uninf.) or infected with the MC1∆*cdtB* (∆*cdtB*) or the MC1TT (TT) strains for 24 h. Activation of the DDR was assessed by immunofluorescence analysis, using antibodies specific for phosphorylated KAP1 (p‐KAP1), phosphorylated CHK2 (p‐CHK2), Ser15 phosphorylated p53 (p‐p53). The upper panel shows representative micrographs of the 1CT cells infected with MC1∆*cdtB* or the MC1TT strains, while the lower panel shows the quantification of positive cells. Mean ± SEM of three to five independent experiments. One hundred cells were evaluated for each experiment for each cell line.
Figure S3. 1CT and 1CTA growth curve
Eighty thousand 1CT and 1CTA cells were plated in 10 cm culture dishes to avoid contact inhibition and left uninfected (Uninf.) or infected with the MC1∆*cdtB* (∆*cdtB*) or the MC1TT (TT) strains. At the indicated time points cells were counted by trypan blue exclusion. Mean ± SEM of five independent experiments. Statistical analysis was performed using the Student *t* test, * p < 0.05
Figure S4. APC deficiency reduces DNA repair and promotes acquisition of genomic instability in murine cells infected with the genotoxic *Salmonella* strain
**A.** *APC* ^*+/+*^ and *APC* ^*+/Min*^ cells were left uninfected (Uninf.) or infected with the MC1 TT (TT) strain for the indicated period of times. Left panel: representative fluorescence micrographs of infected cells illustrating the induction of DNA damage assessed by γH2AX immunofluorescence (green). Nuclei were counterstained with DAPI (blue). Right panel: quantification of the γH2AX positive infected cells. **B.** APC^+/+^ and APC^+/Min^ cells were treated for 6 h with etoposide (ETOP, 15 μM), camptothecin (CPT, 5 μM) or H_2_O_2_ (50 μM) and the percentage of repair was assessed as described in Figure 4, using γH2AX as marker for the DDR. **C.** *APC* ^*+/+*^ and *APC* ^*+/Min*^ cells were infected as described in A, and micronuclei were detected by DAPI staining.
Mean ± SEM of three to four independent experiments. Statistical analysis was performed using the Student's *t* test, * p < 0.05.
Figure S5. Early activation of the DDR response to a broad spectrum of genotoxic stresses
1CT and 1CTA cells, grown in 2D culture, were left untreated (CTR) or treated for 6 h with CDT (1 μg/ml), etoposide (ETOP, 15 μM), camptothecin (CPT, 5 μM) or H_2_O_2_ (50 μM). Activation of the DDR was assessed by immunofluorescence analysis, using antibodies specific for phosphorylated KAP1 (p‐KAP1), phosphorylated H2AX (γH2AX) or 53BP1. Mean ± SEM of three to five independent experiments.
Figure S6. Colony assay
1CTA cells were pretreated with the PI3K inhibitor CDG‐0941 (1 μM) or DMSO as vehicle control for 1 h prior intoxication with CDT (10 ng/ml) in duplicate. Treatment with the inhibitor was repeated every 24 h after intoxication for a week. Colonies were stained with crystal violet 2 weeks post‐intoxication. **A.** Representative picture of the crystal violet staining. **B.** Quantification of the incorporated crystal violet extracted with 2% SDS as described in Experimental procedures. Mean of duplicates.
Figure S7. Colonic organotypic 3D model
**A.** Representative phase contrast micrograph of 1CT cells, grown in 3D culture as described in Material and Methods, stained with hematoxylin and eosin. The white asterisks indicate the presence of colonic fibroblasts embedded in the collagen matrix. **B.** 1CT cells were infected with the MC1 TT strain at the indicated MOI for 24 h. Representative scanning confocal micrographs showing the levels of infection by visualizing the bacteria using a rabbit serum anti‐LPS followed by a donkey anti‐rabbit secondary antibody conjugated to Alexa‐488 (green). Nuclei were counterstained with DAPI (blue). Magnification 40X. **C.** Cells grown in 3D culture were infected with the MC1 ∆*cdtB* or MC1 TT strains at MOI 25:1. Bacteria were visualized using a rabbit serum anti‐LPS followed by a goat anti‐rabbit secondary antibody conjugated to Alexa‐568 (red). Nuclei were counterstained with DAPI (blue). Representative scanning confocal micrographs at magnification 40X.
