# Supplementary material for: Infection with genotoxin‐producing Salmonella enterica synergises with loss of the tumour suppressor APC in promoting genomic instability via the PI3K pathway in colonic epithelial cells
Source: Cell Microbiol. 2019 Aug 26;21(12):e13099. doi: 10.1111/cmi.13099 (PMC6899655; doi:10.1111/cmi.13099)
Supplement: Supplementary file 1 — Figure S1. Phenotypic characterization of APC deficient and proficient cells. A. Representative scanning confocal micrographs showing the staining with TRITC (red)‐ or FITC (green)‐conjugated phalloidin to visualize the actin stress fibers. Nuclei were counterstained with DAPI (blue). Magnification 63X. B. B. Nuclear size was assessed using the ImageJ software from micrograph pictures showed in A. Mean ± SEM of three independent experiments. One hundred cells were evaluated for each experiment for each cell line. Statistical analysis was performed using the Student t test, ** p < 0.01; *** p < 0.001; **** p < 0.0001. Figure S2. Infection with the genotoxic Salmonella strain induces activation of the DDR 1CT and 1CTA cells, grown in 2D culture, were left uninfected (Uninf.) or infected with the MC1∆cdtB (∆cdtB) or the MC1TT (TT) strains for 24 h. Activation of the DDR was assessed by immunofluorescence analysis, using antibodies specific for phosphorylated KAP1 (p‐KAP1), phosphorylated CHK2 (p‐CHK2), Ser15 phosphorylated p53 (p‐p53). The upper panel shows representative micrographs of the 1CT cells infected with MC1∆cdtB or the MC1TT strains, while the lower panel shows the quantification of positive cells. Mean ± SEM of three to five independent experiments. One hundred cells were evaluated for each experiment for each cell line. Figure S3. 1CT and 1CTA growth curve Eighty thousand 1CT and 1CTA cells were plated in 10 cm culture dishes to avoid contact inhibition and left uninfected (Uninf.) or infected with the MC1∆cdtB (∆cdtB) or the MC1TT (TT) strains. At the indicated time points cells were counted by trypan blue exclusion. Mean ± SEM of five independent experiments. Statistical analysis was performed using the Student t test, * p < 0.05 Figure S4. APC deficiency reduces DNA repair and promotes acquisition of genomic instability in murine cells infected with the genotoxic Salmonella strain A. APC +/+ and APC +/Min cells were left uninfected (Uninf.) or infected [file CMI-21-na-s001.pdf]

Figure S1

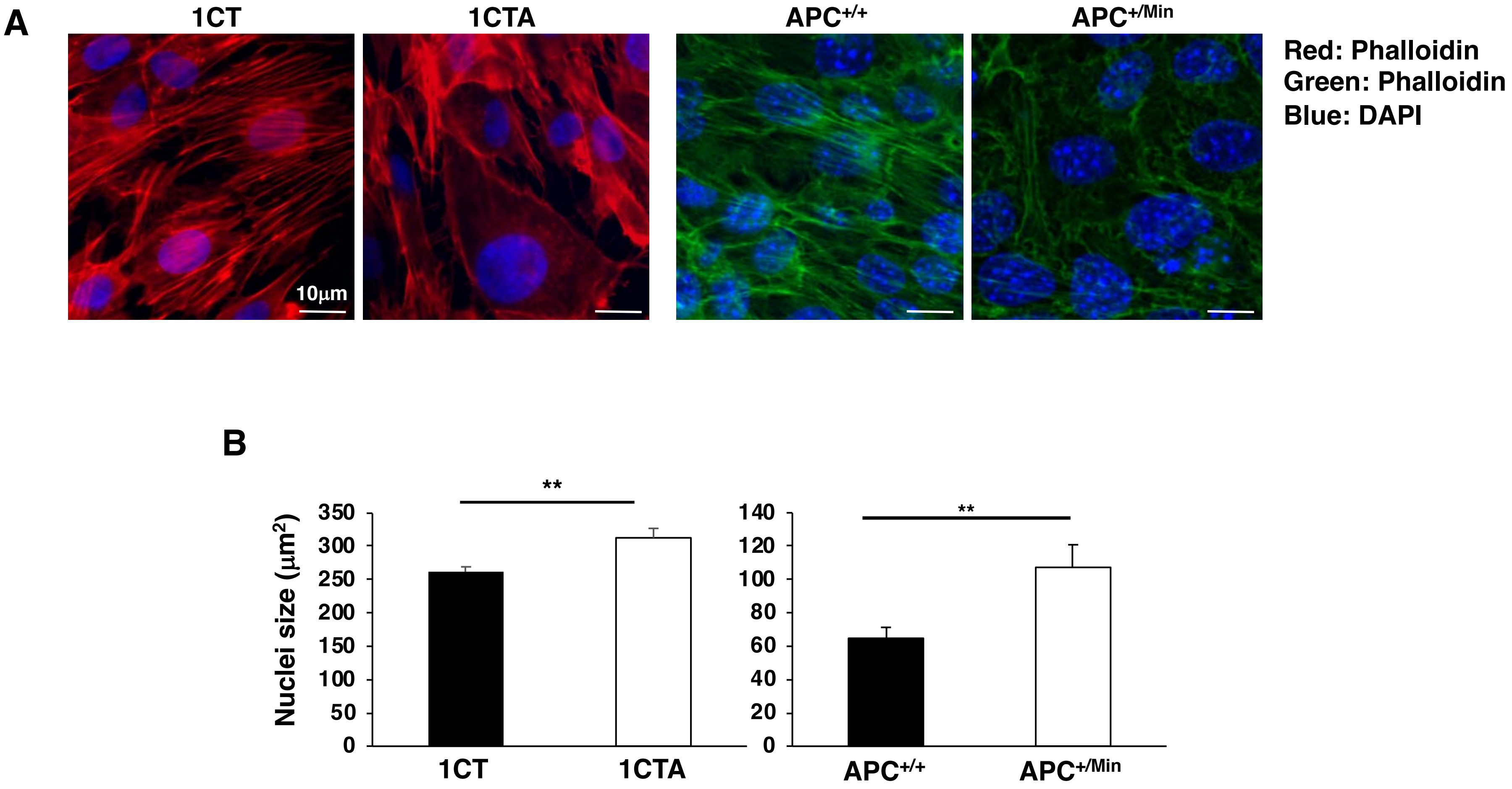
A
1CT
1CTA
APC+/+
APC+/Min
10µm
Red: Phalloidin
Green: Phalloidin
Blue: DAPI
B
**
Nuclei size (µm2)
350
300
250
200
150
100
50
0
1CT
1CTA
140
120
100
80
60
40
20
0
APC+/+
APC+/Min

Figure S2

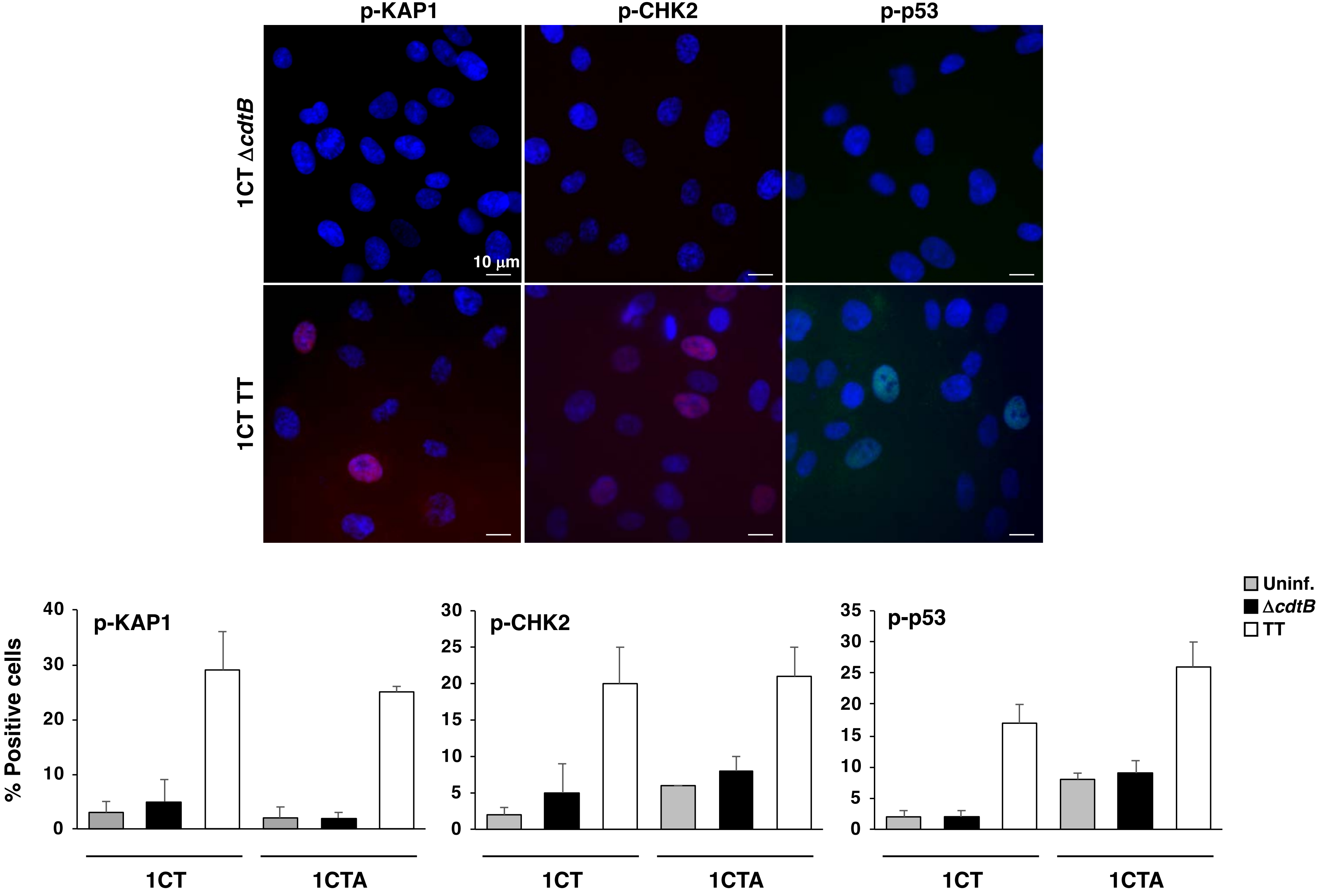
p-KAP1
p-CHK2
p-p53
1CT ΔcdtB
1CT TT
10 μm
Uninf.
ΔcdtB
TT
% Positive cells
p-KAP1
p-CHK2
p-p53
1CT
1CTA
0
5
10
15
20
25
30
35
40

Figure S3

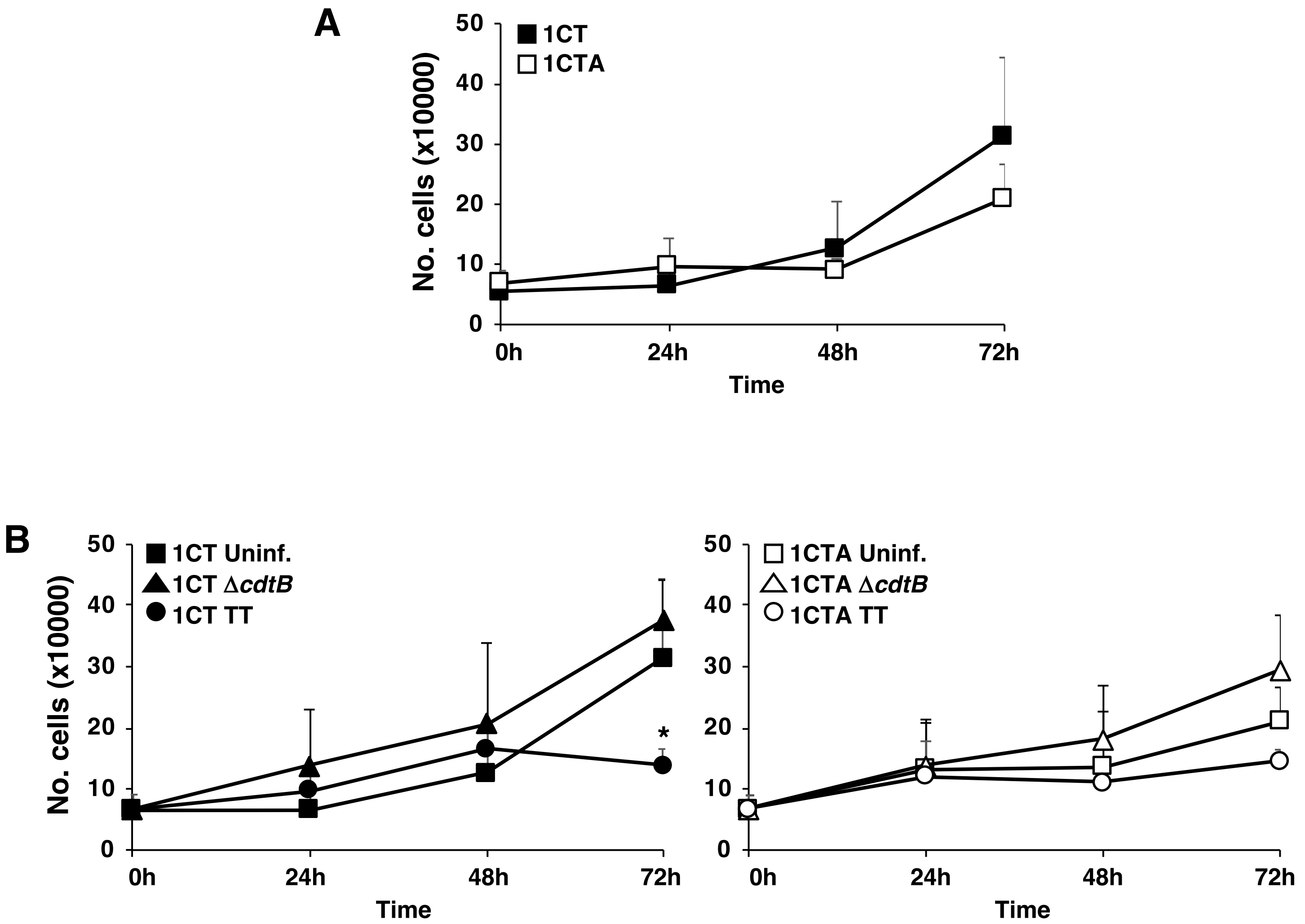
A
1CT
1CTA
No. cells (x10000)
0
10
20
30
40
50
0h
24h
48h
72h
Time
B
1CT Uninf.
1CT ΔcdtB
1CT TT
*
1CTA Uninf.
1CTA ΔcdtB
1CTA TT

Figure S4

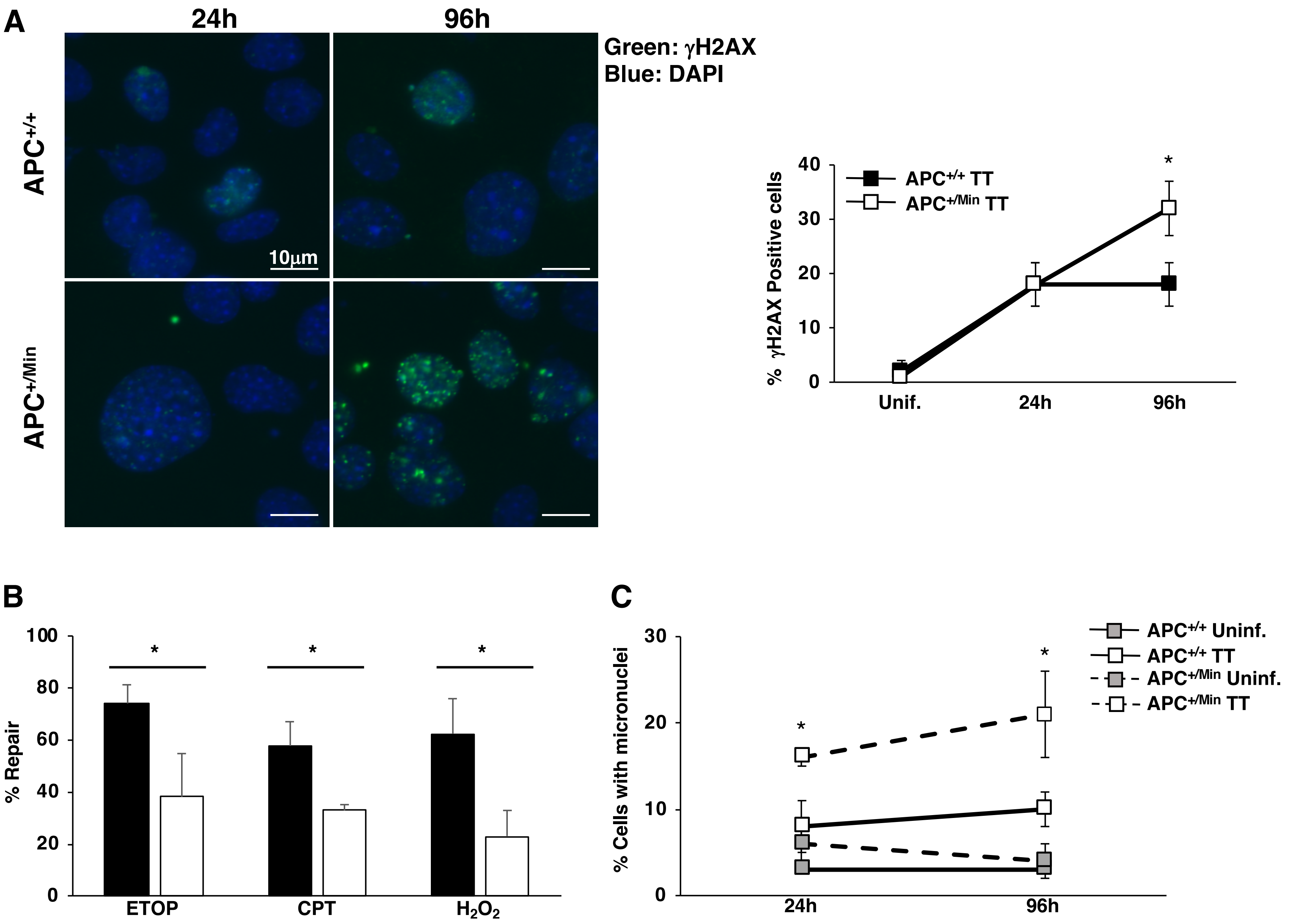
A
24h
96h
Green: γH2AX
Blue: DAPI
APC+/+
APC+/Min
10μm
% γH2AX Positive cells
APC+/+ TT
APC+/Min TT
Unif.
24h
96h
B
% Repair
ETOP
CPT
H2O2
C
% Cells with micronuclei
APC+/+ Uninf.
APC+/+ TT
APC+/Min Uninf.
APC+/Min TT
24h
96h

Figure S5

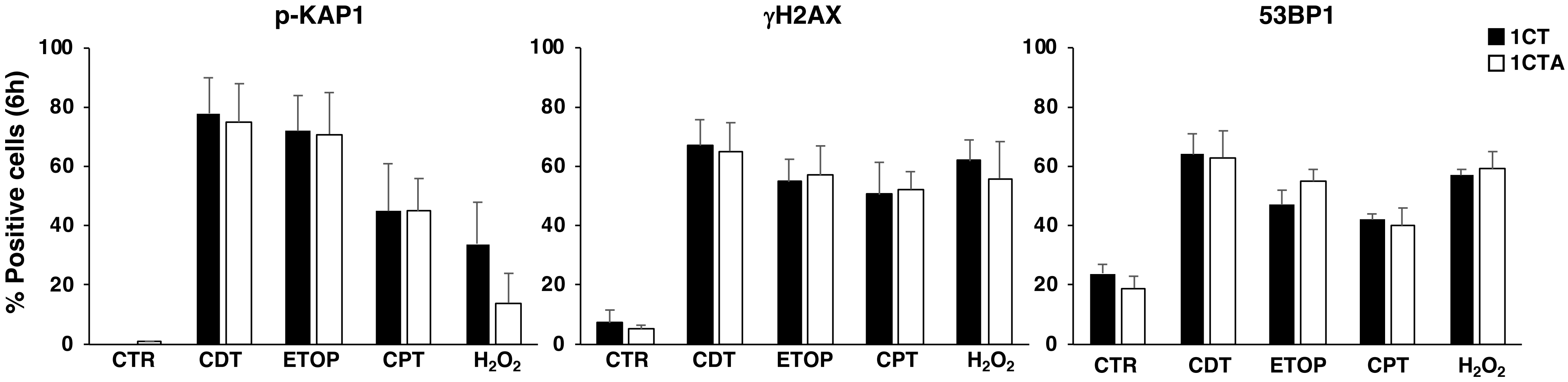

p-KAP1
γH2AX
53BP1
1CT
1CTA
% Positive cells (6h)
100
80
60
40
20
0
CTR
CDT
ETOP
CPT
H2O2

Figure S6

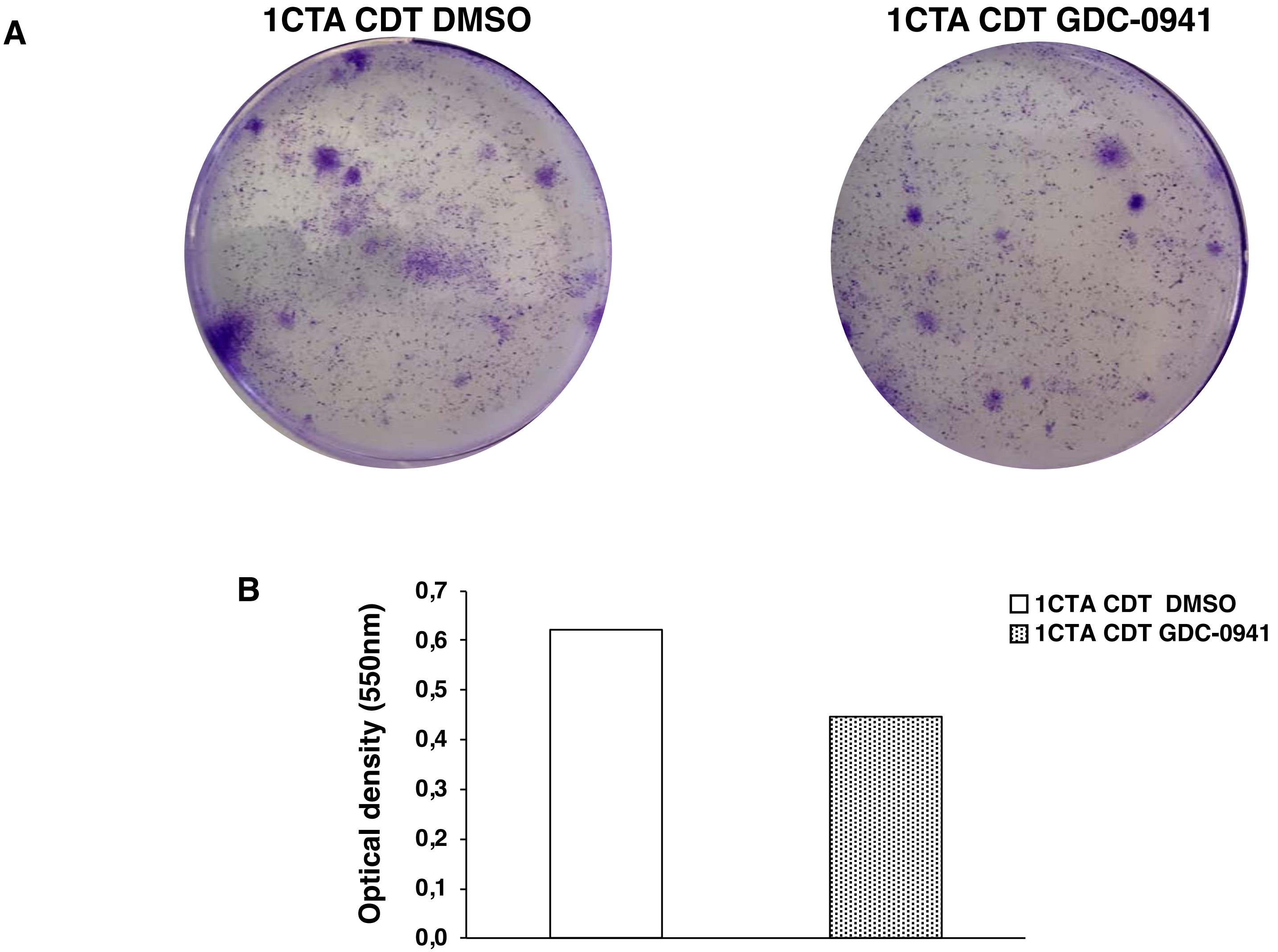
A
1CTA CDT DMSO
1CTA CDT GDC-0941
B
Optical density (550nm)
0,0
0,1
0,2
0,3
0,4
0,5
0,6
0,7
1CTA CDT DMSO
1CTA CDT GDC-0941

Figure S7

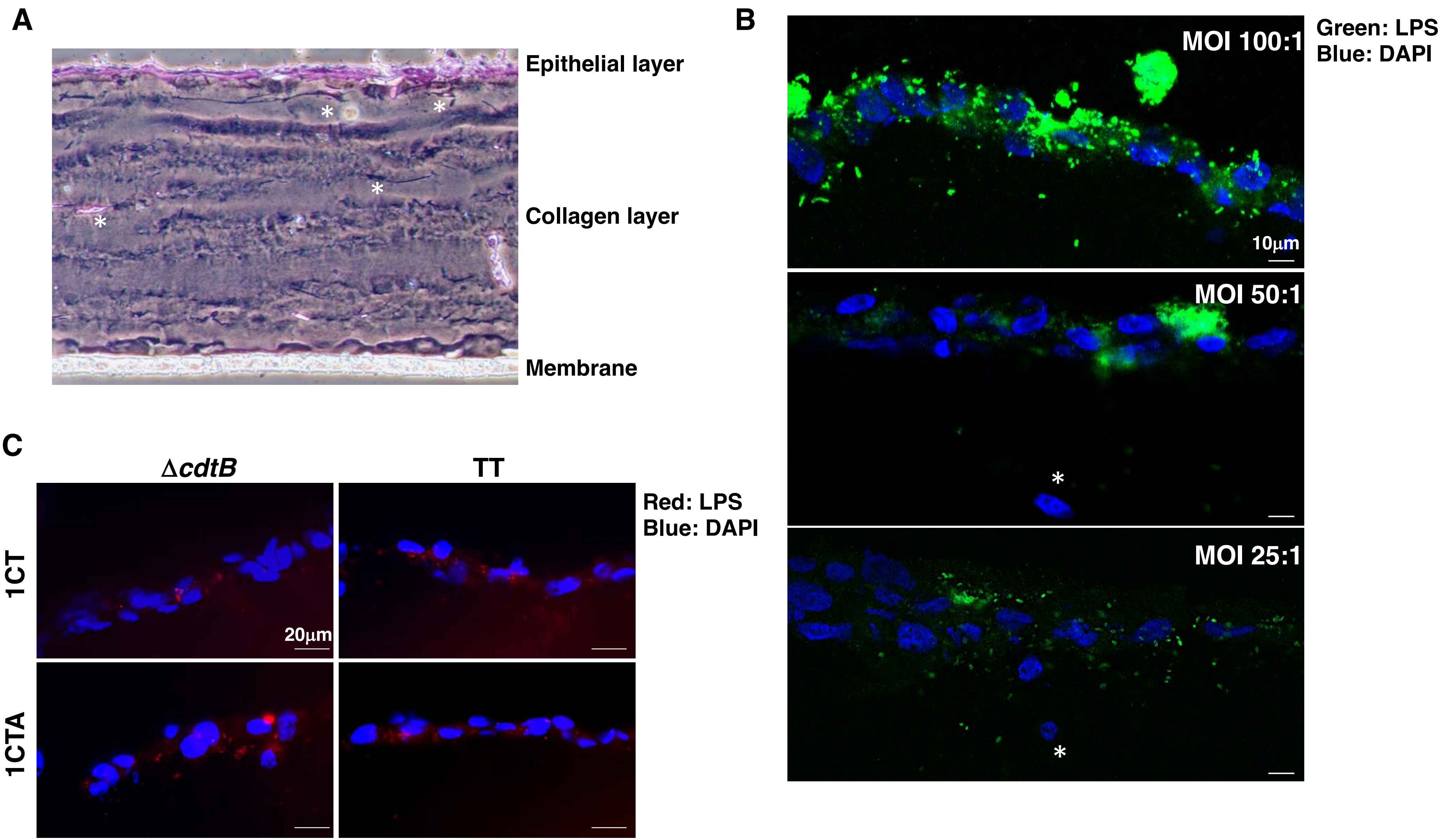

A
Epithelial layer
Collagen layer
Membrane
B
MOI 100:1
Green: LPS
Blue: DAPI
10μm
MOI 50:1
MOI 25:1
C
ΔcdtB
TT
Red: LPS
Blue: DAPI
1CT
1CTA
20μm
